# In-depth Study of a Novel Class of Ditopic Gadolinium(III)-based MRI Probes Sensitive to Zwitterionic Neurotransmitters

**DOI:** 10.3389/fchem.2019.00490

**Published:** 2019-07-24

**Authors:** Ðorđe Toljić, Carlos Platas-Iglesias, Goran Angelovski

**Affiliations:** ^1^MR Neuroimaging Agents, Max Planck Institute for Biological Cybernetics, Tuebingen, Germany; ^2^Centro de Investigacións Científicas Avanzadas, Departamento de Química, Facultade de Ciencias, Universidade da Coruña, A Coruña, Spain

**Keywords:** crown ethers, gadolinium, magnetic resonance imaging, neurotransmitters, zwitterions

## Abstract

The efficacy of Gd-based low-molecular weight ditopic MRI probes on binding zwitterionic neurotransmitters (ZNTs) relies on their structural compatibility. ZNTs are challenging biomarkers for monitoring chemical neurotransmission due to their intrinsic complexity as target molecules. In this work, we focus on tuning the cyclen- and azacrown ether-based binding sites properties to increase the affinity toward ZNTs. Our approach consisted in performing structural modifications on the binding sites in terms of charge and size, followed by the affinity evaluation through *T*_1_-weighted relaxometric titrations. We prepared and investigated six Gd^3+^ complexes with different structures and thus properties, which were found to be acetylcholine insensitive; moreover, two of them displayed considerably stronger affinity toward glutamate and glycine over hydrogencarbonate and other ZNTs. Complexes with small and non-charged or no substituents on the azacrown moiety displayed the highest affinities toward ZNTs, followed by strong decrease in longitudinal relaxivity *r*_1_ of around 70%. In contrast, hosts with negatively charged substituents exhibited lower decrease in *r*_1_ of nearly 30%. The thorough investigations involving relaxometric titrations, luminescence, and NMR diffusion experiments, as well as theoretical density functional theory calculations, revealed that the affinity of reported hosts toward ZNTs is greatly affected by the remote pendant on the azacrown derivative.

## Introduction

A step forward into solving still puzzling physiological and pathological brain conditions would not be possible without a thorough understanding of neural activities. These signaling and metabolic processes consist of a chain of complex interactions and biochemical processes regulated by neurotransmitters, whose imbalance results in numerous neuronal pathologies that permanently damage nervous tissue (Lukasiuk and Pitkänen, 2000; Lewerenz and Maher, 2015). Resolving spatial and temporal concentration change patterns of neurotransmitters released into the synaptic cleft will provide information on the type, localization and degree of occurrence of neuropathological events. Such findings would aid establishing an early diagnosis and allow monitoring of a treatment progress. Therefore, to better understand neuronal events, it is necessary to directly profile neurochemical abnormalities by attempting to understand and assess the diversity in their chemical behavior.

Chemical neural signaling that involves zwitterionic neurotransmitters (ZNTs) in living systems occurs at a sub-second time scale yielding concentrations up to the low milimolar range (Kalivas, 2011). For that reason, the crucial requirement for *in vivo* monitoring besides non-invasiveness and good depth of penetration is high spatio-temporal resolution. These necessities are narrowing down the choice of potential monitoring techniques and due to an effective soft-tissue differentiation, magnetic resonance imaging (MRI) imposes as a modality of choice. An excellent example is the MRI method GluCEST (glutamate-based chemical exchange saturation transfer), which relays on proton exchange between the α-amino group of glutamic acid (Glu) and bulk water (Cai et al., 2012). In practice, this method allows detection of Glu levels across the human brain, which makes it suitable for diagnostic purposes of many pathological states (Roalf et al., 2017). However, it carries intrinsic disadvantages such as signal overlapping with resonances of other metabolites and inability to differentiate between intra and extracellular Glu (Bagga et al., 2018). Thus, one of the ways to overcome these obstacles is imaging living systems more specifically, e.g. via utilization of contrast-enhanced MRI (Lohrke et al., 2016). This approach implies the administration of paramagnetically labeled either extracellular or intracellular sensors able to alter the bulk proton signals upon interaction with targeted metabolites (Cao et al., 2013; Zhang et al., 2017).

ZNTs are rather structurally complex entities as guest molecules. An additional obstacle for their detection is the structural similarity amongst these amino acids, making their differentiation quite difficult. To this end, designing an artificial host capable to selectively recognize and effectively bind ZNTs would require a sophisticated binding platform in terms of structural complexity (Sim and Parker, 2015; Angelovski and Toth, 2017). A notable approach in this direction has been introduced by the Jasanoff lab, which utilized a genetically engineered protein platform that involved a large number of stabilizing interactions (Shapiro et al., 2010). Another approach to sense Glu grounded on the neurotransmitter mimicking strategy has been put forward by the Parker group, aiming to bypass the issues of direct interaction of a probe with Glu (Mishra et al., 2014).

The strategy that allows monitoring the change in longitudinal relaxation time (*T*_1_) which occurs due to host-guest association between the small-size molecule sensors and the ZNTs has been established within the common effort of our collaborators and us (Oukhatar et al., 2015a,b). This approach pioneered the ditopic MR hosts that contain a cavity-like binding pocket suited to simultaneously bind the negatively charged carboxylate and the positively charged α-ammonium cation of amino acid NTs (Steed and Atwood, 2009). The association with guests relies on the anticipated interaction between a coordinatively unsaturated Gd^3+^ with the carboxylate anion and weak hydrogen bonding of a crown ether moiety with the ammonium cation. In these initial studies, it was evidenced that the designed hosts do not distinguish between different ZNTs, making them suitable for functional imaging of neuronal activity that involves excretion of multiple amino acid neurotransmitters.

As a continuation of this study, our investigation focused on the structural optimization of a host binding pocket for sensing the total amount of ZNTs, in specific the most abundant Glu, Gly (glycine), and GABA (γ-aminobutyric acid). The molecular frame of the synthesized complexes consisted of a cyclen-based Gd-chelate as the MR reporting unit covalently linked by a propyl amide linker to the auxiliary 18-azacrown-6 binding site. Moreover, additional efforts were put to optimize the binding sites that would strengthen host-guest interactions. We structurally modified the binding sites, in an attempt to tune the framework to an optimal conformation of the binding pocket and adduct formation. Thus, we varied the size and charge of the substituents on both recognition sites using a set of chemical modifications. We investigated the host-guest binding strength through ^1^H longitudinal relaxivity measurements. Diffusion NMR and fluorescence spectroscopy techniques, as well as density functional theory (DFT) calculations were employed to obtain information at the molecular level. These were performed to highlight the mechanistic and structural aspects responsible for the recognition process, including the changes of the Ln^3+^ coordination environment and the effect of diverse remote groups on the solution structure of the free host and their association with structurally different guests.

## Materials and Methods

### General Remarks

Commercially available reagents and solvents were used without further purification. Procedures for preparation of compounds are provided in the Supplementary Material. Synthesized compounds were purified using silica gel 60 (0.03–0.2 mm) purchased from Carl Roth, Germany. Purification of compounds **11**-**16** was carried out by preparative TLC using Analtech 500 μm thick silica gel GF plates (20 × 20 cm, Uniplate) and visualized under UV light. For the purpose of ^1^H DOSY experiments, ligands **L**^**3**^ and **L**^**5**^ were purified using Preparative HPLC on a Varian PrepStar system equipped with the UV–Vis detector model 335 and a binary pump model SD-1 manual injector, controlled by Star chromatography workstation version 6.3 software. ^1^H, ^13^C, ^31^P, DOSY NMR and *T*_1_ measurements were performed at 298 K on Bruker AVANCE III 300 MHz spectrometer. The NMR spectra were recorded using either CDCl_3_ or D_2_O and referenced to TMS/TSP. Processing was performed using TopSpin 2.1 and the analysis using ACD/SpecManager 9.0 (Advanced Chemistry Development, Inc.). The concentration of the analyzed solutions containing paramagnetic Ln^3+^ ions was determined using the bulk magnetic susceptibility shift (BMS) method (Corsi et al., 2001). The luminescence emission and lifetime measurements were performed on QuantaMaster^TM^ 3 PH fluorescence spectrometer from Photon Technology International, Inc. (USA). Low resolution mass spectra (ESI-MS) were recorded on ion trap SL 1100 Agilent system with an electrospray ionization source. High resolution mass spectra (ESI-HRMS) were performed on a Bruker BioApex II ESI-FT-ICR, equipped with an Agilent ESI source.

### Neurotransmitter Binding Studies

^1^H longitudinal relaxivity titrations of **GdL**^**1−6**^ were performed at 298 K, pH 7.4 (50 mM HEPES) using standard inversion-recovery pulse sequence. The experiments were performed by stepwise addition of analyte to the solution of the analyzed complex (initial concentration of **GdL**^**1−5**^ and **GdL**^**6**^ were 3.0 and 1.0 mM, respectively) and the ^1^H longitudinal relaxation time *T*_1, obs_ was measured after each addition. The longitudinal relaxivity *r*_1_ of bulk water protons was calculated according to Equation 1, where *T*_1, obs_ is the measured value, *T*_1, d_ represents longitudinal relaxation time (diamagnetic contribution) of the medium and [**GdL**] is the milimolar concentration of Gd^3+^.

(1) $$\begin{array}{r} \frac{1}{T_{1,obs}}=\frac{1}{T_{1,d}}+r_{1}\times \left[GdL\right] \end{array}$$

The calculated *r*_1_ values were plotted as a function of added amount of analyte (0–100 equiv.) and fitted to the modified Michaelis-Menten model (**Equation 2**), where the slope of fitted curve is dissociation constant *K*_d_ and [Gd] is the initial concentration of gadolinium complex, while *r*_f_ and *r*_b_ are relaxivities of free and bound complexes, respectively. For the better comparison of host-guest binding strength, inverted values for *K*_d_, the affinity constants *K*_a_, were used.

(2) $$\begin{array}{r} \;y=r_{f}-\left(\frac{r_{f}-r_{b}}{\left[Gd\right]}\right)\times \left(\frac{\left(\left[Gd\right]+x\times \left[Gd\right]+K_{d}\right)-\sqrt{{\left(\left[Gd\right]+x\times \left[Gd\right]+K_{d}\right)}^{2}-4x\times {\left[Gd\right]}^{2}}}{2}\right) \end{array}$$

### Luminescence Measurements

The luminescence measurements with **EuL**^**3, 5**^ (5 mM) were performed in H_2_O and D_2_O (298 K, pH 7.4, 50 mM HEPES) before and after addition 50 equiv. of Gly. The emission spectra were obtained after excitation at 395 nm, while setting both the excitation and emission slit widths at 2 nm bandpass. The decays were measured at the emission wavelength 617 nm with a 10 μs resolution. Excitation and emission slits were set to 5 and 15 nm bandpass, respectively. Data sets are an average of 25 scans and each reported value is the mean of three independent measurements. The obtained curves were fitted to a first-order exponential decay. The *q* values were calculated using modified Horrock's model (**Equation 3**) (Dickins et al., 1996; Beeby et al., 1999), where *n* is the number of NH oscillators of coordinated amide groups.

(3) $$\begin{array}{r} q=1.2\;\times \;\left[\left(k_{H_{2}O}-k_{D_{2}O}\right)-0.25-0.075n\right];n=1 \end{array}$$

### Diffusion Studies

DOSY experiments were performed on a Bruker AVANCE III spectrometer using a BBO probe with z gradients. The samples were dissolved in D_2_O at pD 7.8 and the data were acquired at 298 K. The viscosity correction for the media was performed using the *stebpg1s* pulse sequence and the obtained data were processed using the *T*_1_/*T*_2_ relaxation module. The diffusion measurement on paramagnetic complexes **EuL**^**3, 5**^ were conducted using the standard Bruker pulse sequence *stebpg1s19* employing stimulated echo, bipolar gradients and Watergate solvent suppression. The gradient pulse time was set to 2.5 ms with a diffusion time of 200 ms. The gradient strength was increased linearly over 16 experiments from 2 to 95%. Processing was performed using Bruker Topspin software 2.1. The individual slices of pseudo-2D ^1^H diffusion spectra were phased, base corrected and the diffusion constants were read from the obtained spectra.

(4) $$\begin{array}{r} R_{H}=\frac{kT}{6\pi \eta D} \end{array}$$

The hydrodynamic radii *R*_H_ were calculated using the Einstein-Stokes relation, which models molecules as hydrodynamic spheres (**Equation 4**), where *k* is the Boltzmann constant, *T* is the absolute temperature, η is solvent viscosity and *D* is the measured diffusion coefficient.

### DFT Calculations

All calculations were performed using the Gaussian 09 package (Revision E.01) (Frisch et al., 2009) within the hybrid meta-generalized gradient approximation (hybrid meta-GGA) with the TPSSh exchange-correlation functional (Tao et al., 2003). All calculations used the large-core quasi-relativistic effective core potential (LCRECP) approximation developed by Dolg and the corresponding [5s4p3d]-GTO valence basis set for Gd (Dolg et al., 1989), whereas the ligand atoms were described by using te standard 6–31G(d,p) basis set. The stationary points found on the potential-energy surfaces as a result of geometry optimizations were confirmed to correspond to energy minima rather than saddle points by using frequency analysis. Solvent effects were included by using the integral-equation formalism variant of the polarizable continuum model (IEFPCM) (Tomasi et al., 2005). Relative energies include zero point energy (ZPE) corrections obtained with frequency calculations.

## Results and Discussion

### Design of Ditopic Hosts GdL^1−6^

**Figure 1:**
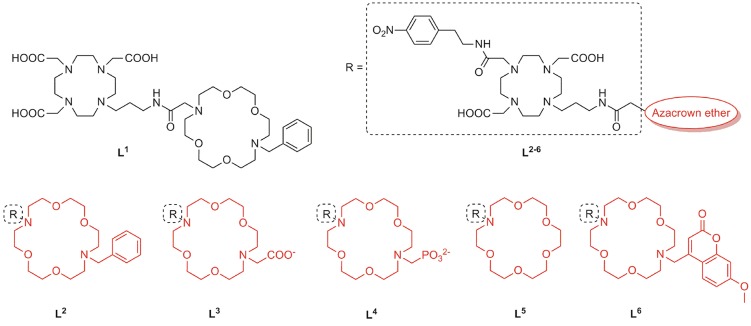
Chemical structures of the ligands **L**^**1−6**^ for the preparation of ditopic molecular hosts.

We implemented a diversity-oriented synthetic strategy to prepare a set of probes with structurally different pendant arms at both binding sites with the aim to intensify the interaction between the NH${}_{3}^{+}$ group of amino acid NTs and the free electronic pairs of the azacrown ether heteroatoms. To this end, we prepared six different Gd^3+^ complexes which differ in the structure of the MR reporting unit or the azacrown ether (Figure 1). Thus, **L**^**1**^ was designed by combining the common Gd-chelator based on DO3A (1,4,7,10-tetraazacyclododecane-1,4,7-tricarboxylic acid) with the diazacrown ether that bears a charge-neutral benzyl (Bn) group, whereas the remaining **L**^**2−6**^ contain a DO2A moiety (1,4,7,10-tetraazacyclododecane-1,7-dicarboxylic acid) as Gd-chelator and azacrown ethers with substituents of different charge and size **L**^**2−4, 6**^ or without substituent **L**^**5**^. The rationale behind design of **L**^**1, 2**^ lies on tuning the charge of the fragment containing the Gd^3+^ complex, which should impact the binding of ZNTs through their carboxylate group. The non-charged and small Bn substituent was expected to allow a facile approach of an ammonium cation. Ligands with negatively charged substituents, an acetate in **L**^**3**^ and a phosphonate in **L**^**4**^, were designed to exchange multiple bifurcated hydrogen bonds of an NH${}_{3}^{+}$ unit and increase the contribution of electrostatic interactions.

In the second approach, we designed **L**^**5, 6**^ to explore the effect of the substituent size on the conformational organization of the binding pocket, which is crucial for the recognition of the guest. Our previous studies evidenced that the carbonyl group of the propyl-amide linker is weakly coordinated to Gd^3+^, causing cyclen-crown sites to adopt a half opened shell-like conformation, which is well-suited for binding an amino acid functional groups (Oukhatar et al., 2015a,b). Arising from this, we designed **L**^**5**^ with no substituent in an attempt to favor strong amide linker coordination. On the contrary, a sterically bulky substituent methyl-7-methoxycoumarin (Mmc) was attached in the host **L**^**6**^, which could theoretically disturb this interaction due to increased steric constrains. Moreover, the strong fluorescence emission properties of the Mmc group enable additional interaction studies by means of the fluorescence spectroscopy. Last but not least, by combining different types of MR chelators (DO3A or DO2A) and substituents on the azacrown ethers, we set out to investigate the influence of the net charge of the complexes on their affinity toward the ZNTs.

### Synthesis of Complexes LnL^1−6^

**Scheme 1:**
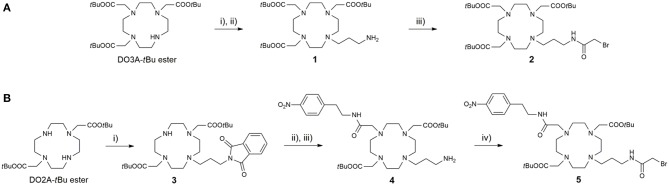
Synthesis of **(A)** DO3A-derived and **(B)** DO2A-derived bromide fragments **2** and **5**, respectively. Reagents and conditions: **(A)** (i) *N*-(bromopropyl)phthalimide, MeCN, K_2_CO_3_, 45°C; (ii) *i*-PrOH, EDA, 60°C, 75% (over two steps); (iii) CH_2_Cl_2_, DCC, bromoacetic acid, 75%; **(B)** (i) *N*-(bromopropyl)phthalimide, MeCN, NaHCO_3_, 3 days, 65%; (ii) 2-bromo-*N*-(4-nitrophenyl)acetamide, MeCN, K_2_CO_3_; (iii) *i*-PrOH, EDA, 65% (over two steps); (iv) CH_2_Cl_2_, DCC, bromoacetic acid, 52%.

The preparation of ligands **L**^**1−6**^ relies on the convergent synthetic strategy, linking cyclen-derived bromides with 18-crown-6 ether fragments via direct N-alkylation. The synthesis of bromides **2** and **5** is summarized in Scheme 1. The bromo fragment **2** was prepared in 4 steps, starting from cyclen. In the first step, cyclen was converted in DO3A *tert*-butyl ester in a one-step procedure (Machitani et al., 2008), followed by the subsequent N-alkylation with *N*-(3-bromopropyl)phtalimide and the mild deprotection of Phth with ethylenediamine (EDA) to afford the propyl amine derivative **1**. The amine **1** was coupled with bromoacetic acid in the presence of *N, N*'-dicyclohexylcarbodiimide (DCC) to furnish bromide **2**. In a similarly fashion, the DO2A *tert*-butyl ester was synthesized in 3 steps according to a previously published procedure (Kovacs and Sherry, 1997). This chelator precursor was first functionalized with the Phth-protected propyl amine linker to yield **3**. Subsequently, the second aromatic linker was introduced in the cyclen ring in the *trans* position relative to the propyl linker, and the Phth group was cleaved to yield the amine **4**, which was subjected to the same treatment as the DO3A analog to give the desired DO2A-derived bromide **5**.

**Scheme 2:**
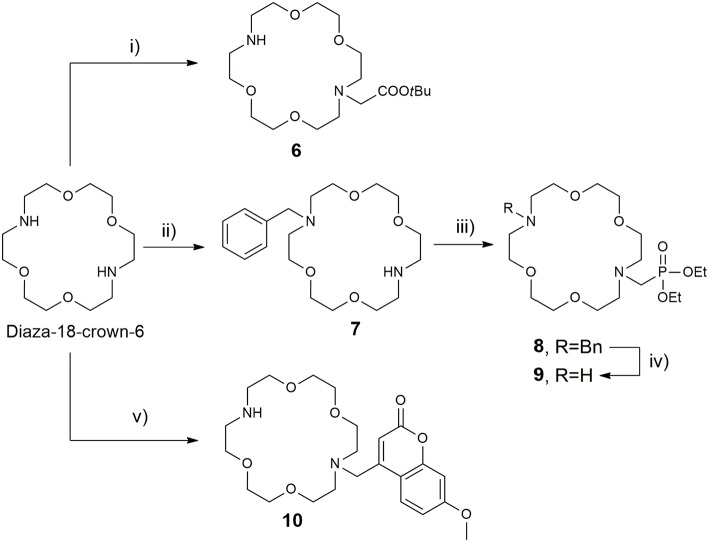
Synthesis of 18-azacrown-6-ether fragments **6, 9**, and **10**. Reagents and conditions: (i) *tert*-butyl bromoacetate (0.95 equiv), CHCl_3_, Et_3_N, RT, 48 h, 62%; (ii) BnCl (0.95 equiv), CHCl_3_, Et_3_N, RT, 60 h, 58%; (iii) (C_2_H_5_O)_2_P(H)O, CH_2_O, CH_2_Cl_2_, BF_3_·Et_2_O, anh. Na_2_SO_4_, 65°C, 60%; (iv) H_2_, Pd(OH)_2_/C, EtOH, 3 bar, 94%; (v) Br-Mmc, MeCN, NaHCO_3_, RT, 40%.

The synthesis of the azacrown fragments **6, 7, 9**, and **10** commenced from the common precursor 1,10-diaza-18-crown-6-ether, attaching substituents via direct N-alkylation with the corresponding alkyl halides, *tert*-butyl bromacetate, benzyl bromide, and Br-Mmc, respectively Scheme 2. The adduct **8** was generated under the Kabachnik-Fields reaction conditions, followed by the catalytic debenzylation in a Parr hydrogenator to afford the final mono *N*-phosphonated derivative **9**.

**Scheme 3:**
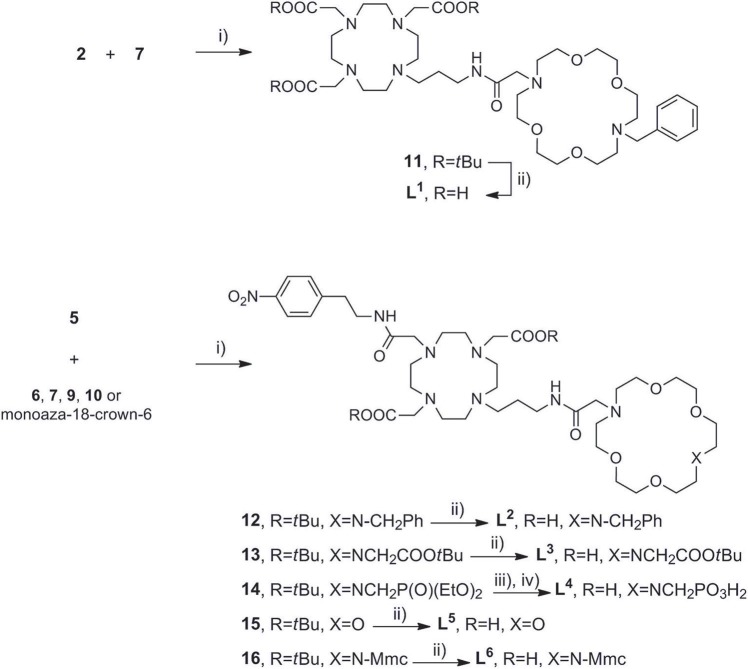
Synthesis of **(A)** DO3A derived ligand **L**^**1**^, **(B)** DO2A derived ligands **L**^**2−6**^. Reagents and conditions: **(A)** (i) dry DMF, N_2_, NaHCO_3_, 3 days, 70%; (ii) HCO_2_H, overnight, RT, 76%. **(B)** (i) dry DMF, N_2_, NaHCO_3_, 3 days, 25–78%; (ii) HCO_2_H, 32 h, 45°C; (iii) TMBS (16 equiv), dry DCM, N_2_; (iv) H_2_O, 50%.

Once the precursors of two binding sites were prepared, they were conjugated into the *bis*-macrocyclic constructs via direct N-alkylation Scheme 3. In general, bridging bromides **2** and **5** with the azacrown ether fragments required over 3 days stirring in dry DMF and N_2_ atmosphere to acquire **11–16** in acceptable to good yields (25–75%), due to slow kinetics and tendency of bromides to undergo hydrolysis. Subsequently, the obtained *tert*-butyl esters **11–13, 15**, and **16** were hydrolyzed with formic acid at 60°C over 24 h into the final ligands **L**^**1−3, 5, 6**^. Deprotection of **14** required first transesterification with BrSiMe_3_, followed by hydrolysis of trimethylsilyl esters to give **L**^**4**^. The purity of ligands **L**^**1−6**^ was determined with respect to nitrogen content obtained in CHN analysis and the amount of corresponding LnCl_3_ salt for complexation was calculated accordingly in stoichiometric ratio of 1:1. Finally, the complexation of **L**^**1−6**^ was performed in water, maintaining pH ~ 7.2 at 60°C for 16 h to ensure formation of the thermodynamically favored cyclen in-cage coordination and yield the final paramagnetic complexes.

### Relaxometric Studies

The relaxometric efficacy of the synthesized complexes **GdL**^**1−6**^ was evaluated in *T*_1_-titration experiments with the three most abundant ZNTs: Gly, Glu and GABA, the non-zwitterionic neurotransmitter acetylcholine (ACh) and the competitive bicarbonate anion. As well-established, the main contributing factor to the ^1^H longitudinal relaxivity *r*_1_ is the hydration state *q* of the paramagnetic label (Merbach et al., 2013). Thus, upon addition of the analyte (A), the hydrated chelate **GdL(H**_**2**_**O)**_***q***_ associates into the ternary adduct **GdL(A)**, in which the coordinated water molecule(s) are displaced to a different extent. This results in longer longitudinal relaxation times (*T*_1_) of bulk water protons, that is, reduced longitudinal relaxivity *r*_1_. Furthermore, plotting the *r*_1_ values vs. the amount of the added analyte and fitting the obtained curves to a modified Michaelis-Menten mathematical model provides the affinity constants for the formed ternary complexes.

**Figure 2:**
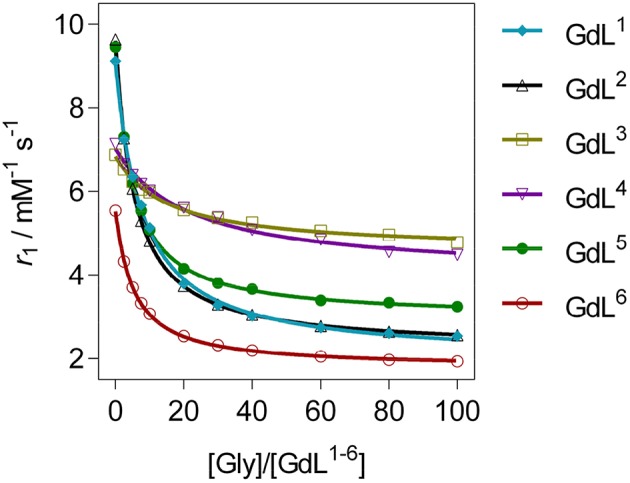
^1^H longitudinal relaxometric titration curves for **GdL**^**1−6**^ with Gly ([**GdL**^**1−5**^] = 3.0 mM, pH 7.4 or [**GdL**^**6**^] = 1.0 mM, pD 7.8, 50 mM HEPES, 298 K, *B*_0_ = 7.05 T). The lines represent the fits as explained in the text.

The *r*_1_ profiles of **GdL**^**1−6**^ upon the titration with Gly revealed that patterns in relaxometric response are associated with the nature of substituents at the recognition moieties (Figure 2). More specifically, the observed downward trends are controlled by the size and charge of the pendant arm at the auxiliary receptor, according to which complexes were classified in three groups, whilst the dominant factor that determined affinity is found to be the coordination environment on the reporting moiety (Table 1). Specifically, the first group of complexes consists of those which bear the non-charged and sterically small benzyl substituent **GdL**^**1, 2**^ or no substituent **GdL**^**5**^ at the auxiliary moiety. The features that characterize this group are very high initial *r*_1_ values that exceeded well over 9.0 mM^−1^ s^−1^, and a decrease upon saturation with Gly to 30, 31, and 36% of the starting *r*_1_ value, respectively. The second group of complexes are those containing negatively charged pendants **GdL**^**3, 4**^, which displayed relatively high initial relaxivities, 6.88 and 7.14 mM^−1^ s^−1^, respectively. However, the relaxivity drop until the saturation point is halved when compared with the first group: addition of Gly lowered the initial *r*_1_ by 30 and 37%, respectively. The last complex **GdL**^**6**^, characterized by the sterically bulky and non-charged coumarin group Mmc, exhibited the lowest initial relaxivity of 6.08 mM^−1^ s^−1^, but a comparable decrease in *r*_**1**_ with **GdL**^**1, 2, 5**^ of 63% when saturated with Gly.

**Table 1:** Binding affinity constants and relaxivity decrease for complexes **GdL**^**1−6**^ upon saturation with Gly ^[^^a^^]^^[^^b^^]^.

**Complexes**	**GdL^**1**^**	**GdL^**2**^**	**GdL^**3**^**	**GdL^**4**^**	**GdL^**5**^**	**GdL^**6**^**
*K_*a*_*[M^−1^]	47	68	23	16	76	69
–Δ*r*_1_[%]	70	69	26	31	64	63

a *K_a_ were obtained by fitting ^1^H NMR relaxometric titrations curves according to Equation 2*.

b *r_1_ decrease after saturation with 60 equiv. of Gly*.

**Figure 3:**
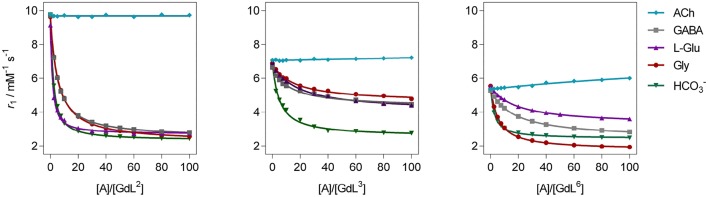
^1^H longitudinal relaxometric titration curves for **GdL**^**2, 3, 6**^ with different analytes **A** ([**GdL**^**2, 3**^] = 3.0 mM or [**GdL**^**6**^] = 1.0 mM, 298 K, pH 7.4, 50 mM HEPES, *B*_0_ = 7.05 T). The lines represent the fits as explained in the text.

Regarding the affinities toward Gly, the investigated complexes showed slightly different trends with respect to their amplitudes of *r*_**1**_ change (Table 1, Figure 3, and Figure S1). The highest affinities falling in the range 69–75 M^−1^ were observed for **GdL**^**2, 5, 6**^, the complexes that bear Gd-DO2A as the MR reporting unit and no or non-charged Bn and Mmc substituents on the azacrown ether moiety. The DO3A analog of **GdL**^**2**^, namely complex **GdL**^**1**^, exhibited a lower *K*_a_ value, indicating that shifting the net charge of the MR reporting moiety from positive to neutral logically reduced the affinity toward the negatively charged carboxylate group of the ZNTs. On the other hand, the negatively charged substituents on the azacrown ether side in the two DO2A-derived complexes **GdL**^**3, 4**^ did not compensate for their size and potentially additional, undesired interaction with the paramagnetic center (*vide infra*). Indeed, this group of complexes exhibited the lowest affinities, whereby the more negatively charged phosphonate group influenced **GdL**^**4**^ to show decreased affinity toward Gly to the greater extent and the lowest *K*_a_ value of all investigated systems.

In terms of specificity, it is found that the hosts **GdL**^**1, 2**^ displayed greater affinity toward Glu over other ZNTs and hydrogencarbonate giving values 72 and 311 M^−1^, respectively, while all the synthesized complexes were ACh-insensitive (Table S2). The improved affinity of **GdL**^**1**^ and **GdL**^**2**^ toward Glu over the other ZNTs is probably related to the longer chain length of the neurotransmitter combined to the favorable effect of the Bn pendant, which introduces low steric hindrance and forces the 18-crown-6 moiety into a conformation that is more suited to accommodate the ammonium cation. It was also noticed that **GdL**^**6**^ exhibited a higher affinity toward sterically less demanding Gly over GABA and Glu. On contrary, the observed diversity in affinity patterns for DO2A derivatives with different substituents on the azacrown ether moiety suggested the synergistic effect of several factors on the recognition process. More specifically, the milder downward trends and lesser change in *r*_1_ for **GdL**^**3, 4**^, as well as the lower initial relaxivity for **GdL**^**6**^, indicated that either increase in remote substituent size or the net negative charge have a major impact on binding ZNTs.

### Luminescence Studies

**Figure 4:**
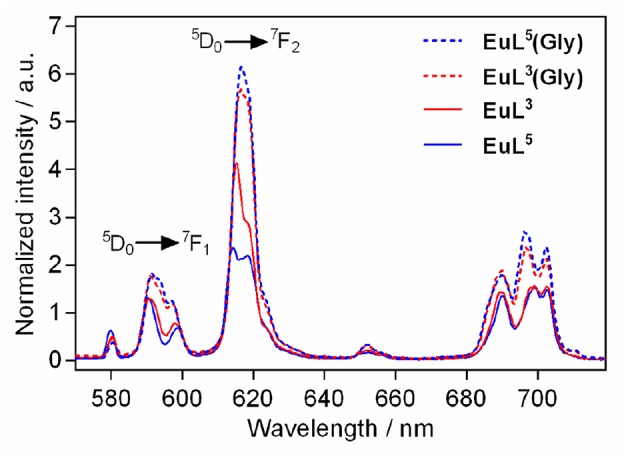
Metal-centered luminescence emission spectra for **EuL**^**3, 5**^ before and after addition 50 equiv. of Gly (λ_ex_ = 395 nm, [**EuL**^**3, 5**^] = 5 mM, pH 7.4, 50 mM HEPES, 298 K).

We performed luminescence studies on **EuL**^**3**^ and **EuL**^**5**^ to examine their solution structures and the way that auxiliary pendants affect binding with ZNTs (Binnemans, 2015). In the first step, the luminescence emission spectra of both complexes before and upon addition of 50 equiv. Gly were measured. The **EuL**^**3**^**(H**_**2**_**O)**_***q***_ complex showed more intense emission than **EuL**^**5**^**(H**_**2**_**O)**_***q***_, but this situation is reversed upon saturation with Gly, with the most intense emission being observed for the ternary adduct **EuL**^**5**^**(Gly)** (Figure 4). The investigations carried out on similar host-systems evidenced that association of ZNTs with Eu^3+^ labeled complexes induce hyperchromicity of the hypersensitive ^5^D_0_→^7^F_2_ transition at ca. 615 nm (Oukhatar et al., 2015b). The intensity increase is related with the coordination of negatively charged carboxylate that displaces the water molecule coordinated directly to Eu^3+^, thus reducing the quenching efficiency of the Eu^3+^ ^5^D_0_ state by water O-H oscillators (Beeby et al., 1999). Therefore, we next measured the radiative rate constants characterizing the depopulation of the ^5^D_0_ excited state in H_2_O and D_2_O solutions and the hydration numbers were calculated for both hydrated complexes **EuL**^**3, 5**^**(H**_**2**_**O)**_***q***_ and their adducts **EuL**^**3, 5**^**(Gly)** (Dickins et al., 1996). The calculated *q* value for **EuL**^**5**^**(H**_**2**_**O)**_***q***_ yielded 1.76, which is twice higher than that of **EuL**^**3**^**(H**_**2**_**O)**_***q***_; furthermore, the addition of Gly expectedly decreased the hydration numbers of both complexes (Table 2). The greater drop (Δ*q* = 0.87) occurred for **EuL**^**5**^, pointing out that H_2_O is displaced more readily than in **EuL**^**3**^, in which change in hydration is almost negligible. The hydration number of **EuL**^**5**^ suggests the presence of two water molecules coordinated to the metal ion, which would imply that the oxygen atom of the propylamide group is not involved in coordination to the metal ion. This is in contrast with previous findings when investigating structurally related systems (Oukhatar et al., 2015b). These results indicate that slight modifications of the ligand structure may have an important impact in the structure of the complexes.

**Table 2:** Luminescence lifetimes of Eu^3+^ measured at 616 nm from the decay profile of the ^5^D_0_ excited state (H_2_O and D_2_O) and calculated hydration numbers *q* ^[^^a^^]^.

**Complex**	**No Gly**			**+** **50 eq. Gly**			
	**_^τ^H_2_O^[ms]^_**	**_^τ^D_2_O^[ms]^_**	***q***	**_^τ^H_2_O^[ms]^_**	**_^τ^D_2_O^[ms]^_**	***q***	**Δ*q***
**EuL**^**3**^	0.51	1.11	0.88	0.52	1.07	0.80	0.08
**EuL**^**5**^	0.4	1.43	1.76	0.58	1.51	0.89	0.87

a *[**EuL**^**3,5**^] = 5 mM, pH 7.4, 50 mM HEPES, 298 K*.

The metal-centered emission spectra revealed diverse coordination environments for the hydrated forms of the complexes (Binnemans, 2015). Namely, **EuL**^**3**^**(H**_**2**_**O)**_***q***_ exhibited larger intensity ratio of the magnetic-dipole transition ^5^D_0_→^7^F_1_, which is insensitive of the local coordination environment, to the hypersensitive ^5^D_0_→^7^F_2_ transition, which is heavily influenced by the local symmetry of Eu^3+^ and the nature of ligand atoms (Table S3). These data suggested a solution form for **EuL**^**3**^**(H**_**2**_**O)**_***q***_ in which a more polarizable ligand atom than water oxygen is coordinated to Eu^3+^, partly inhibiting the binding of Gly. Relying on these observations, we hypothesized that the acetate pendant of the lariat ether fragment in **EuL**^**3**^**(H**_**2**_**O)**_***q***_ interacts weakly in intra- or intermolecular fashion with the paramagnetic label, favoring a form in which the lanthanide ion is shielded, and thus impedes water coordination. If this is the case, the more polarizable Gly-carboxylate ligand replaces the azacrown ether-acetate pedant by disrupting this interaction, which should induce major conformational changes in **EuL**^**3**^ when Gly is added. However, the exchange of the ligand acetate group by Gly in the coordination sphere of Eu^3+^ excludes water involvement, resulting in the small changes observed by time-resolved emission decay measurements.

Furthermore, **EuL**^**6**^ appended with the coumarine derived fluorophore, was characterized prior and upon the interaction with Gly by means of fluorescence spectroscopy. However, the complex turned out to be non-emissive when the metal center was excited directly or via the chromophore. Moreover, the fluorophore on **EuL**^**6**^ exhibited strong fluorescence emission intensity at 420 nm when excited at 330 nm, typical for coumarine derivatives (Figure S2). The steady-state fluorescence emission experiments were repeated also in the presence of Gly or Glu, resulting in spectra of similar intensity as with **EuL**^**6**^ alone. Hence, this methodology could not provide further evidences related to interaction of the 18-azacrown-6 ether moiety with NH${}_{3}^{+}$ groups of ZNTs.

### Diffusion Studies

**Figure 5:**
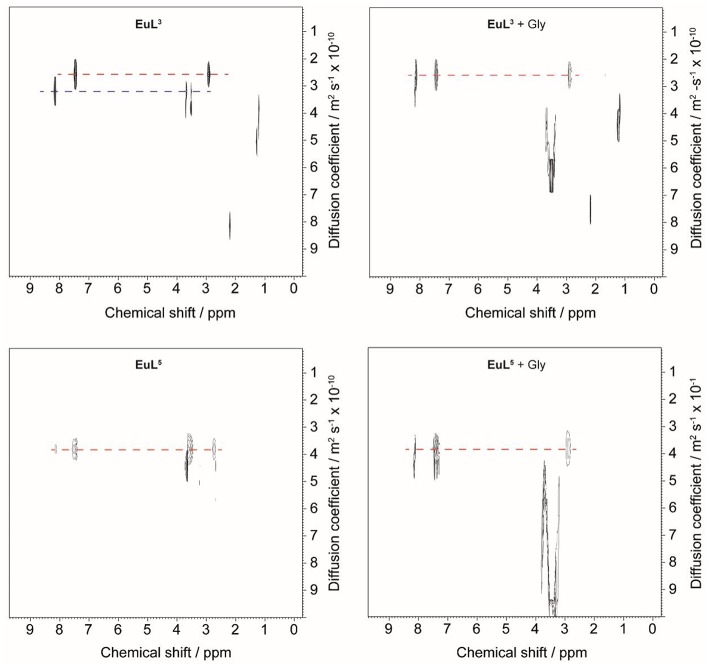
^1^H DOSY NMR of **EuL**^**3**^ (top) and **EuL**^**5**^ (bottom) in D_2_O before (left) and after (right) addition of 50 equiv. Gly.

Diffusion-ordered 2D NMR spectroscopy (DOSY) allows separation of NMR signals based on the molecular diffusion. Owing to the correlation between self-diffusion and the size/shape of molecular species, represented by Stokes-Einstein model, ^1^H DOSY-NMR is a readily used technique to disperse ^1^H resonances along the diffusion axis, relating signals with differently organized forms of a solute (Morris et al., 1994). To verify the hypothesis brought upon luminescence measurements, we performed ^1^H NMR diffusion measurements on paramagnetic complexes **EuL**^**3, 5**^ and their ternary adducts **EuL**^**3, 5**^**(Gly)**, and compared the calculated hydrodynamic radii (*R*_H_) assuming spherical shape of species (Table 3) (Natrajan et al., 2007; Maggini et al., 2012; Denis-Quanquin et al., 2016). Firstly, the ^1^H DOSY-NMR experiments on aqueous solution of complexes **EuL**^**3**^ and **EuL**^**5**^ were recorded (Figure 5). The obtained pseudo 2D spectra for **EuL**^**3**^**(H**_**2**_**O)**_***q***_ identified two distinct sets of ^1^H resonances with corresponding different diffusion constants (*D*), while the **EuL**^**5**^**(H**_**2**_**O)** complex resonated along one diffusion value. Following this step, the diffusion measurements were repeated after 50 equiv. of Gly were added, revealing disappearance of the resonances corresponding to the faster diffusing form in **EuL**^**3**^**(Gly)**.

**Table 3:** Diffusion coefficients obtained in ^1^H DOSY NMR experiments and calculated hydrodynamic radii ([**EuL**^**3, 5**^] = 10 mM, D_2_O as solvent, pD = 7.8, 25°C) ^[^^a^^]^^[^^b^^]^.

	**EuL^**3**^**	**EuL^**3**^ + Gly**	**EuL^**5**^**	**Eu^**5**^ + Gly**
Diffusion coefficient *D* (10^−10^ m^2^ s^−1^)	2.57 3.19^[^^c^^]^	2.57	3.81	3.81
Hydrodynamic radius *R*_H_ (nm)	1.91 1.54^[^^c^^]^	1.91	1.29	1.29

a *Conditions: [***EuL***^**3, 5**^] = 10 mM, D_2_O as a solvent, pD 7.8, 298 K, B_0_ = 7.05 T*.

b *Hydrodynamic radii R_H_ are calculated according to Equation (4)*.

c *Values for the faster diffusing form of hydrated complex **EuL**^**3**^*.

**Figure 6:**
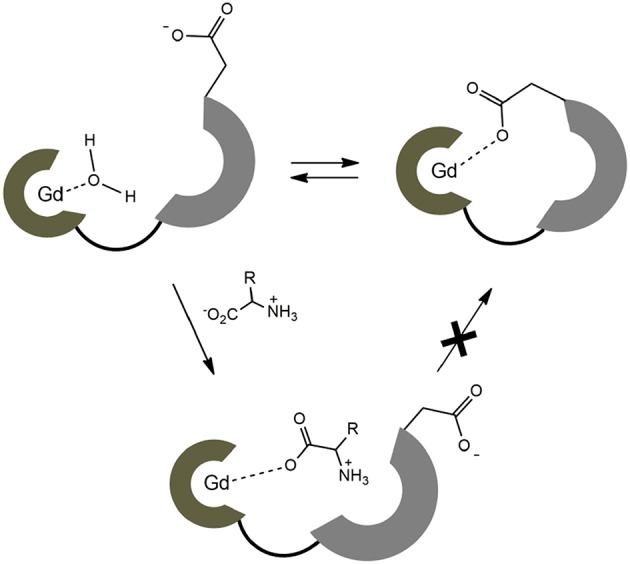
Schematic presentation of dynamic equilibrium for the hydrated complex **EuL**^**3**^ and the proposed binding mechanism with ZNTs.

On the contrary, no changes in diffusion of the slower diffusing form in **EuL**^**3**^**(Gly)** or diffusion of **EuL**^**5**^**(Gly)** were observed (Figure 5). These results indicate that two solution forms of **EuL**^**3**^**(H**_**2**_**O)**_***q***_ are in dynamic equilibria, in which the form with smaller hydrodynamic radius underwent structural changes in the presence of Gly. Transformation of this form into one with the greater hydrodynamic radius, likely eliminates the probability of **EuL**^**3**^**(H**_**2**_**O)**_***q***_ intermolecular self-organization into dimeric and oligomeric forms. Instead, the formation of **EuL**^**3**^ species with smaller diameter that disappear with Gly addition rather suggests a weak intramolecular self-assembly, i.e., coordination of the azacrown acetate pendant to Eu^3+^. This interaction brings closer the two binding moieties toward each other, thus reducing *R*_H_. Once Gly approaches the coordination sphere of the paramagnetic metal ion, the acetate pendant is displaced from Eu^3+^ with the carboxylate of Gly, allowing formation of ternary complex **EuL**^**3**^**(Gly)** (Figure 6).

These observations are in good agreement with the results obtained in the relaxometric titrations and luminescence emission experiments (*vide supra*): existence of the dynamic equilibrium of non-folded and folded forms in **EuL**^**3**^ and **GdL**^**3**^ results in lower initial hydration number *q* and consequently lower initial *r*_1_ value prior any interaction with Gly (or ZNT in general). In such case, the observed *r*_1_ changes after addition of Gly occur only due to the displacement of coordinated water on the species without the intramolecular self-assembly. In contrast to the **GdL**^**3**^ system, **GdL**^**5**^ exists only in the non-folded conformation; here, the Gly/ZNTs addition causes greater change in hydration number and hence greater drop in *r*_1_.

### DFT Calculations

**Figure 7:**
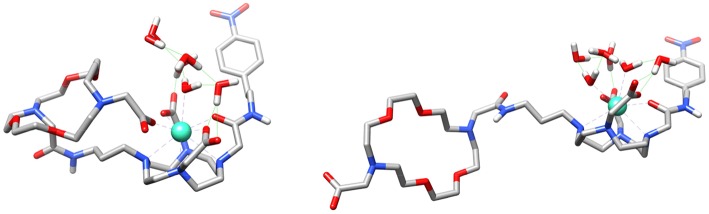
Structures of the **GdL**^**3**^**(H**_**2**_**O)**_**2**_·**4H**_**2**_**O** system obtained with DFT calculations showing the coordination of the carboxylate group of the crown moiety to the metal ion (left) and the open form (right).

Theoretical calculations were carried out to rationalize the different behavior of the **GdL**^**3**^ and **GdL**^**5**^ complexes and provide support to the interpretation of relaxometric, luminescence and DOSY experiments. We first analyzed whether the involvement of the carboxylate group attached to the crown moiety in **GdL**^**3**^ in intramolecular coordination to the metal ion is feasible. We thus modeled the **GdL**^**3**^**(H**_**2**_**O)**_**2**_·**4H**_**2**_**O** system, which included two water molecules coordinated to the metal ion and four additional second-sphere water molecules. The explicit inclusion of a few second-sphere water molecules was done because it was previously found to be essential for providing a good description of the Gd-O_water_ distances and the spin densities at the ^17^O nucleus (Esteban-Gomez et al., 2012; Regueiro-Figueroa and Platas-Iglesias, 2015). The calculation provided an energy minimum that contained two water molecules coordinated to the Gd^3+^ ion, which presents the typical capped square antiprismatic (SAP) coordination environment of this type of complexes. Next, we explored the potential energy surface by approaching the remote carboxylate group to the metal ion, which eventually leads to a second energy minimum in which the carboxylate group coordinates to Gd^3+^ by replacing a coordinated water molecule (Figure 7). Indeed, these calculations indicated that the coordination of the remote carboxylate group is feasible. Moreover, DFT predicts a very small energy difference between the two energy minima (folded and non-folded species), favoring the open form by only 1.4 kJ mol^−1^. Calculations performed on the **GdL(H**_**2**_**O)·5H**_**2**_**O** system (Figure S12) also suggest that coordination of the phosphonate group attached to the crown moiety is likely responsible for the lower binding affinity of this complex toward ZNTs.

**Figure 8:**
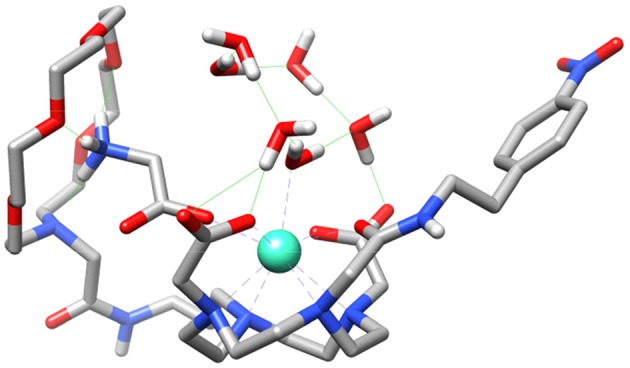
Structure of the **GdL**^**5**^**(Gly)(H**_**2**_**O)·5H**_**2**_**O** system obtained with DFT calculations showing the simultaneous binding of Gly to the crown moiety and the Gd^3+^ ion.

We also performed calculations on the **GdL**^**5**^**(H**_**2**_**O)**_**2**_·**4H**_**2**_**O** and **GdL**^**5**^**(Gly)(H**_**2**_**O)·5H**_**2**_**O** systems to gain insight into the molecular recognition process. The calculations were done using three model systems with the Gly guest interacting with either the crown moiety or the lanthanide ion, and a third form in which simultaneous recognition by the two binding units is taking place (Figure 8 and Figures S10, S11). The corresponding energy differences provided an estimate of the contributions to the overall recognition process of the coordination to the metal ion and the hydrogen bonding interaction with the crown moiety, which turned out to be 17.3 and 9.7 kJ mol^−1^. Thus, these calculations indicate that binding to the coordinatively unsaturated GdDO2A unit represents the major driving force of the molecular association process, though the hydrogen-bonding interaction with the crown moiety also provides a sizeable contribution to the overall binding energy.

## Conclusions

In the present work, we have expanded the scope of Gd^3+^ based DO3A/DO2A ditopic MR probes sensitive to zwitterionic neurotransmitters (ZNTs) and revealed chemical structure-affinity relationships through the relaxometric response. The results indicated that the lanthanide coordination environment is highly sensitive to influences of the pedant arms on the azacrown ether moieties, resulting in significant differences on both initial relaxivity and the total relaxivity change. Furthermore, the charge neutral DO3A derivative **GdL**^**1**^ displayed reduced affinity over the DO2A analog **GdL**^**2**^, while adding the negative charge on the azacrown ethers reduced the affinity toward guests. Indeed, it is found that the relaxometric response of all derivatives with the same MR reporting unit **GdL**^**2−6**^ is governed by the nature of the auxiliary pendant. More specifically, the substituent at the azacrown ether fragment favors the disposition of binding sites in a charge- and size-dependent manner, thus determining both relaxivity change and binding affinity of the paramagnetic host. The observed *r*_**1**_ patterns revealed that increasing in size of the pendant elevates steric hindrances, resulting in lower initial relaxivities while only slightly diminishing the strength of interactions with guest molecules. Besides steric hindrance, electronic factors in the complexes with negatively charged pendants **GdL**^**3, 4**^ unfavored host-guest association to the greater extent, likely due to the coordination of the remote carboxylate/phosphonate groups to the metal ion. In addition, an achievement of great importance evidenced here is that **GdL**^**1−2**^ displayed higher affinity toward Glu over competitive hydrogencarbonate. The findings reported in this work present a breakthrough in understanding the nature of interactions between the ditopic MR platforms consisted of DO3A-/DO2A-derived units and azacrown ethers with ZNTs. Owning the ability to produce an excellent change in relaxivity, these MR sensors present a great potential for further development of bioresponsive probes that can visualize changes in concentration of the most abundant ZNTs by means of *T*_1_-weigthed MRI. Additionally, the results of this study can be used as guideline for development of novel concepts within functional MRI for sensing chemical neurotransmission.

## Data Availability

The summary of data is available in the standard reporting form, while the raw data supporting the conclusions of this manuscript will be made available by the authors. Requests to access the datasets should be directed to goran.angelovski@tuebingen.mpg.de.

## Author Contributions

GA designed the targeted molecules and supervised research. ÐT synthesized and evaluated the compounds, analyzed the data, wrote the manuscript. CP-I performed DFT calculations and wrote the section concerning computational calculations. All authors critically reviewed the manuscript.

### Conflict of Interest Statement

The authors declare that the research was conducted in the absence of any commercial or financial relationships that could be construed as a potential conflict of interest.
