# Supplementary material for: In-depth Study of a Novel Class of Ditopic Gadolinium(III)-based MRI Probes Sensitive to Zwitterionic Neurotransmitters
Source: Front Chem. 2019 Jul 24;7:490. doi: 10.3389/fchem.2019.00490 (PMC6668053; doi:10.3389/fchem.2019.00490)
Supplement: Supplementary file 1 [file Data_Sheet_1.pdf]

Supplementary Material

In-depth Study of a Novel Class of Ditopic Gadolinium(III)-based MRI Probes Sensitive to Zwitterionic Neurotransmitters

Đorđe Toljić,[1] Carlos Platas-Iglesias[2] and Goran Angelovski[1,*]

[1] Đ. Toljić, Priv.-Doz. Dr. G. Angelovski
MR Neuroimaging Agents
Max Planck Institute for Biological Cybernetics
Max-Planck-Ring 11, 72076 Tübingen, Germany
E-mail: goran.angelovski@tuebingen.mpg.de

[2] Prof. Dr. C. Platas-Iglesias
Centro de Investigacións Científicas Avanzadas (CICA) and Departamento de Química, Facultade de Ciencias, Universidade da Coruña
15071 A Coruña, Spain

Contents

1. Experimental details of ligand synthesis....S2
2. Relaxometric studies....S16
3. Luminescence studies....S17
4. NMR Spectra of ligands **L**$^{1-6}$....S18
5. DFT calculations....S24
6. References....S44

1. Experimental details of ligand synthesis

Synthetic procedures

[4-(3-Amino-propyl)-7,10-bis-*tert*-butoxycarbonylmethyl-1,4,7,10tetraaza-cyclododec-1-yl]-acetic acid *tert*-butyl ester (1)

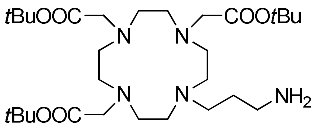

K_2CO_3 (1.69 g, 12.12 mmol) was added to a stirred solution of DO3A *tert*-butyl ester (2.4 g, 4.66 mmol) in MeCN (20 mL) at room temperature, followed by portionwise addition of *N*-(-3-bromopropyl)phthalimide (1.66 g, 6.06 mmol) over 1 h. Upon completed addition, the mixture was vigorously stirred at 60 ^{o}C for 16 hours. The mixture was then filtered and the filtrate was evaporated under the reduced pressure to dryness. Thereafter, the remaining residue was redissolved in *i*-PrOH, followed by the addition of EDA (2.52 mL, 37.3 mmol). The temperature of reaction mixture was raised to 60 ^{o}C and the mixture was stirred for 4 h. The mixture was then concentrated in vacuo and the obtained dark yellow residue was purified by the column chromatography (silica gel, MeOH/CH_2Cl_2, 4:96) to afford **1** (1.7 g 65 %) as an off-white solid: R_f = 0.68 (MeOH/CH_2Cl_2, 15:85); **^{1}H NMR** (300 MHz, $CDCl_3$): δ (ppm) 3.43-3.08 (br, 8H, CH_2), 2.89-2.30 (br, 16H, CH_2), 1.63 (quint, 2H, CH2CH_2CH2), 1.55-1.39 (overlapped s, 27H, C(CH_3)$_3$); **^{13}C NMR** (75 MHz, $CDCl_3$): δ (ppm) 172.3, 170.7, 82.0, 81.9, 58.0, 57.2, 56.7, 50.7, 50.6, 50.5, 50.2, 50.0, 39.6, 28.0, 23.8; **LRMS (ESI-TOF)** *m/z* $[M + H]^+$ calcd. for $C_{29}H_{58}N_5O_6^+$ 572.4, found 572.4.

{4-[3-(2-Bromo-acetylamino)-propyl]-7,10-bis-*tert*-butoxycarbonylmethyl-1,4,7,10tetraaza-cyclododec-1-yl}-acetic acid *tert*-butyl ester (2)

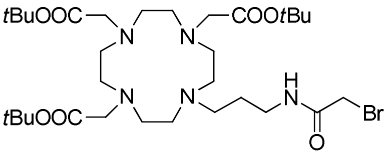

The compound **2** was synthesized according to the previously published procedure.(Angelovski et al., 2008) The analytical data matched those previously reported.

{7-*tert*-Butoxycarbonylmethyl-4-[3-(1,3-dioxo-1,3-dihydro-isoindol-2-yl)-propyl]-1,4,7,10tetraaza-cyclododec-1-yl}-acetic acid *tert*-butyl ester (3)

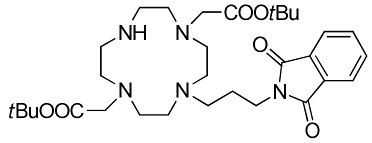

$NaHCO_3$ (0.483 g, 5.59 mmol) was added to a stirred solution of DO2A *tert*-butyl ester (2.4 g, 5.99 mmol) in MeCN (60 mL) at room temperature, followed by portionwise addition of *N*-(-3-bromopropyl)phthalimide (1.45 g, 5.39 mmol) over 4 hours. Upon completed addition, the mixture was vigorously stirred for 3 days. The mixture was then filtered and the filtrate was evaporated under the reduced pressure to dryness. The solid residue was then dissolved in CH_2Cl_2 (60 mL) and washed with water (2 x 25 mL). The organic layer was dried over anh. Na_2SO_4 and evaporated under the vacuo. The resulting dark yellow resin was washed with low amount of cooled Et_2O to give **4** (2.1 g. 62 %) as an off-white solid. **^{1}H NMR** (300 MHz, $CDCl_3$)**:** δ (ppm) 7.92-7.66 (m, 4H, PhthC*H*), 3.70 (t, *J* = 7.1 Hz, 2H, C*H*$_2$NPhth), 3.33 (br s, 4H, NC*H*$_2$CO$_2$*t*Bu), 3.09 (br m, 4H, NC*H*$_2$CH$_2$N), 2.92 (br s, 4H, NHCH$_2$C*H*$_2$N), 2.81 (br s, 4H, NHC*H*$_2$CH$_2$N), 2.76-2.55 (br, 6H, CH$_2$C*H*$_2$CH$_2$ and NCH$_2$C*H*$_2$N), 1.78 (quint, 2H, CH$_2$C*H*$_2$CH$_2$), 1.45 (br s, 18H, C(C*H*$_3$)$_3$); **^{13}C NMR** (75 MHz, $CDCl_3$): δ (ppm) 170.4, 168.3, 134.2, 131.8, 123.3, 81.5, 58.1, 51.1, 50.9, 49.0, 47.7, 43.6, 36.2, 28.1, 21.1; **HRMS (ESI-TOF)** *m/z* $[M + H]^+$ calcd. for $C_{31}H_{50}N_5O_6^+$ 588.3756, found 588.3761.

2-Bromo-*N*-[2-(4-nitro-phenyl)-ethyl]-acetamide

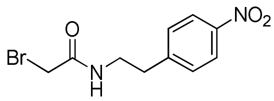

DIPEA (2.45 mL, 14.06 mmol) was added to a stirred suspension of 2-(4-nitrophenyl)ethylamine hydrochloride (3 g, 14.06 mmol) in CH_2Cl_2 (10 mL). The resulting mixture was cooled down to 0 ^oC and the bromoacetic acid (2.32 g. 16.17 mmol) dissolved in CH_2Cl_2 (10 mL) was added dropwise. Then the coupling reagent EDC (3.21 g, 16.17 mmol) and the additive HOBt (2.55 g, 16.17 mmol) were added and the resulting mixture was stirred at room temperature for 2 hours. The crude reaction mixture was cooled down, filtered and washed with 2N HCl solution (3 x 25 mL) and brine (2 x 20 mL). The organic phase was collected, dried over anh. Na_2SO_4 and the solvent was evaporated under reduced pressure to

yield **3** as an off-white solid (3.25 g, 80 %). The analytical data matched those previously reported for the same compound (Gündüz et al., 2015).

(4-(3-Amino-propyl)-7-*tert*-butoxycarbonylmethyl-10-{[2-(4-nitro-phenyl)-ethylcarbamoyl]-methyl}-1,4,7,10tetraaza-cyclododec-1-yl)-acetic acid *tert*-butyl ester (4)

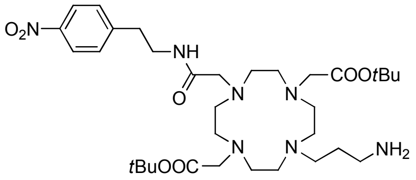

K_2CO_3 (1.2 g, 8.58 mmol) was added to a solution of **4** (2.1 g, 2.65 mmol) in dry MeCN and the mixture was stirred for 30 min. at room temperature. The compound **3** (1.24 g, 4.29 mmol) was then added portionwise, the temperature was elevated to 45 °C and left to stir for 16 hours. Thereafter, the reaction mixture was cooled down, solvent was removed under the vacuum and the remaining residue was redissolved in *i*-PrOH followed by the addition of EDA (1.43 mL, 21.6 mmol). The temperature of reaction mixture was raised to 60 °C and the mixture was stirred for 4 h. Then the mixture was concentrated in vacuo and the obtained dark yellow residue was purified by the column chromatography (silica gel, $MeOH/CH_2Cl_2$, 5:95) to give **5** (1.2 g, 65 %) as an off-white solid: R_f = 0.31 ($MeOH/CH_2Cl_2$, 10:90); **^{1}H NMR** (300 MHz, $CDCl_3$): δ (ppm) 8.11 (d, J = 7.55 Hz, 2H, ArH), 7.58 (d, J = 7.93 Hz, 2H, ArH), 3.71-1.94 (br, 30H, CH_2), 1.84-1.35 (overlapped s, 20H, $CH_2CH_2CH_2$ and $C(CH_3)_3$); **^{13}C NMR** (75 MHz, $CDCl_3$): δ (ppm) 172.0, 170.2, 147.9, 146.4, 130.2, 123.4, 82.0, 58.7, 56.6, 56.4, 56.3, 50.6, 50.2, 49.5, 40.0, 38.8, 34.8, 28.0, 23.8; **HRMS (ESI-TOF)** *m/z* $[M + H]^+$ calcd. for $C_{33}H_{58}N_7O_7^+$ 664.4392, found 664.4398.

(4-[3-(2-Bromo-acetylamino)-propyl]-7-*tert*-butoxycarbonylmethyl-10-{[2-(4-nitro-phenyl)-ethylcarbamoyl]-methyl}-1,4,7,10tetraaza-cyclododec-1-yl)-acetic acid *tert*-butyl ester (5)

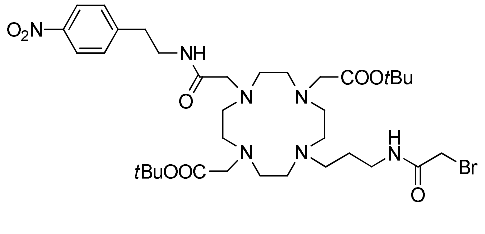

Bromoacetic acid (0.404 g, 2.82 mmol) and DCC (0.587 g, 2.82 mmol) were subsequently added as solids to a stirred solution of the amine **5** (1.1 g, 1.66 mmol) in CH_2Cl_2 (30 mL) and the reaction mixture was stirred at room temperature for 8 h. Then, the precipitate was filtered off and the filtrate was concentrated under the reduced pressure. The crude residue was purified by the column chromatography (silica gel, $MeOH/CH_2Cl_2$ gradient from 5:95 to 30:70) to yield **6** (840 mg, 52 %) as an off-white *-+solid: R_f = 0.5 ($MeOH/CH_2Cl_2$, 15:85); **^{1}H NMR** (300 MHz, $CDCl_3$): δ (ppm) 8.62 (m, 1H, *H*NCO), 8.10 (d, *J* = 8.69 Hz, 2H, ArH), 7.67 (m, 1H, *H*NCO), 7.48 (d, *J* = 8.69 Hz, 2 H), 3.95 (br s, 2H, CH_2Br), 3.65-2.07 (br, 30H, CH_2), 1.74 (quint, 2H, $CH_2CH_2CH_2$), 1.48 (br s, 18 H, $C(CH_3)_3$); **^{13}C NMR** (75 MHz, $CDCl_3$): δ (ppm) 172.8, 171.1, 166.3, 147.9, 146.4, 130.0, 123.4, 82.7, 56.7, 56.3, 53.5, 51.6, 51.1, 50.9, 50.1, 39.7, 38.3, 35.3, 29.5, 28.0, 25.2; **HRMS (ESI-TOF)** *m/z* $[M + H]^+$ calcd. for $C_{35}H_{59}BrN_7O_8^+$ 784.3603, found 784.3596.

(1,4,10,13-Tetraoxa-7,16-diaza-cyclooctadec-7-yl)-acetic acid *tert*-butyl ester (6)

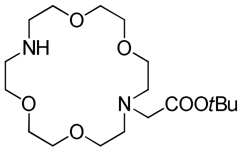

The solution of *tert*-butyl bromoacetate (0.32 mL, 2.13 mmol) in $CDCl_3$ (25 mL) was added dropwise to a stirred mixture of 4,13-diaza-18-crown 6-ether (0.59 g, 2.24 mmol) and Et_3N (0.32 mL, 2.24 mmol) in $CDCl_3$ (25 mL) over 8 h and the resulting mixture was stirred at room temperature for 2 days. Then the reaction mixture was evaporated under the vacuum and the residue was purified by the column chromatography (silica gel, 3.5 % $MeOH/CH_2Cl_2$) to give **10** (0.52 g, 62 %) as a pale yellow solid: R_f = 0.23 ($MeOH/CH_2Cl_2$, 15:85); **^{1}H NMR** (300 MHz, $CDCl_3$): δ (ppm) 4.03-3.81 (m, 4H, $HNCH_2CH_2O$), 3.71-3.47 (overlapped m, 12H, OCH_2CH_2N and OCH_2CH_2O), 3.42-3.33 (overlapped s, 2H, $NCH_2C(O)O$), 3.21-3.06 (br m, 4 H, NCH_2CH_2O), 3.03-2.90 (m, 4 H, OCH_2CH_2N), 1.49-1.41 (overlapped s, 9 H, $C(CH_3)_3$); **^{13}C NMR** (75 MHz, $CDCl_3$): δ (ppm) 170.4, 81.4, 70.2, 69.9, 67.8, 66.5, 53.9, 50.8, 48.7, 28.3; **HRMS (ESI-TOF)** *m/z* $[M + H]^+$ calcd. for $C_{18}H_{37}N_2O_6^+$ 377.2646, found 377.2651.

7-Benzyl-1,4,10,13-tetraoxa-7,16-diaza-cyclooctadecane (7)

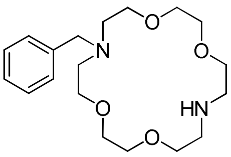

Et_3N (0.32 mL, 4.48 mmol) was added to a stirred solution of 4,13-diaza-18-crown 6-ether (1.0 g, 3.74 mmol) in $CDCl_3$ (25 mL) and the mixture was stirred for 30 min. Benzyl chloride (0.43 mL, 3.74 mmol) in $CDCl_3$ (25 mL) was then added dropwise over 8 h and the resulting mixture was stirred at room temperature for 2 days. Then the solvent was removed under the reduced pressure and the solid residue was purified by the column chromatography (silica gel, 5 % $MeOH/CH_2Cl_2$) to give compound **7** (0.79 g, 60 %) as an off-white solid: R_f = 0.16 ($MeOH/CH_2Cl_2$, 15:85); **^{1}H NMR** (300 MHz, $CDCl_3$): δ (ppm) 7.40-7.19 (overlapped m, 5H, Ar*H*), 3.88 (t, 4H, $NHCH_2CH_2O$), 3.68 (br s, 2 H, CH_2Ph), 3.66-3.43 (overlapped m, 12H, OCH_2), 3.17 (t, 4H, $NHCH_2CH_2$), 2.77 (t, *J* = 4.91 Hz, 4H, NCH_2CH_2); **^{13}C NMR** (75 MHz, $CDCl_3$): δ (ppm) 138.5, 128.9, 128.2, 126.9, 70.0, 69.9, 68.6, 66.5, 57.8, 55.1, 48.4; **HRMS (ESI-TOF)** *m/z* $[M + H]^+$ calcd. for $C_{19}H_{33}N_2O_4^+$ 353.2435, found 353.2438.

(16-Benzyl-1,4,10,13-tetraoxa-7,16-diaza-cyclooctadec-7-ylmethyl)-phosphonic acid diethyl ester (8)

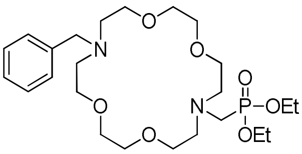

Formaldehyde (48 mg, 1.59 mmol) and anh. Na_2SO_4 (250 mg) were added to a solution of diethyl phosphonate (218 µL, 1.59 mmol) in dry CH_3Cl (15 mL) and the resulting mixture was stirred for 30 min. at room temperature. Then the compound **7** (350 mg, 0.99 mmol) was added followed by the addition of $BF_3 \cdot Et_2O$ (10 µL) and the reaction mixture was refluxed for 6 h. The reaction mixture was then cooled down to r.t, filtered off, the filtrate was concentered in vacuo and the obtained solid residue was purified by the column chromatography (silica gel, $MeOH/CH_2Cl_2$ gradient from 4:96 to 8:92) to afford product **8** (298 mg, 60 %) as a dark yellow solid: R_f = 0.42 ($MeOH/CH_2Cl_2$, 20:80); **^{1}H NMR** (300 MHz, $CDCl_3$): δ (ppm) 7.52-7.14 (overlapped m, 5H, ArH), 4.13 (quint, 4H, CH_2CH_3), 3.80 (br s, 2H, NCH_2Ph), 3.74-3.49 (overlapped m, 16H, OCH_2CH_2), 3.17-2.50 (overlapped m, 10H, CH_2CH_2N and $NCH_2P(O)$, 1.34 (t, *J* = 7.08 Hz, 6H, CH_3); **^{13}C NMR** (75 MHz, $CDCl_3$): δ (ppm) 139.2, 129.3, 128.3, 127.3, 70.6, 69.9, 69.3, 69.3, 61.9, 61.8, 59.6, 55.3, 55.2, 53.5,

51.9, 49.8, 16.6, 16.5; **^{31}P NMR** (121.5 MHz, $CDCl_3$): δ (ppm) 25.46; **HRMS (ESI-TOF)** *m/z* $[M + Na]^+$ calcd. for $C_{24}H_{43}N_2NaO_7P^+$ 525.2700, found 525.2707.

(1,4,10,13-Tetraoxa-7,16-diaza-cyclooctadec-7-ylmethyl)-phosphonic acid diethyl ester (9)

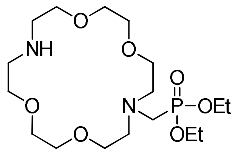

The catalyst $Pd(OH)_2/C$ (20 % w/w) was suspended in the solution of **8** (298 mg, 0.59 mmol) in EtOH (25 mL) and catalytic amount of NH_4OH (50 µL, 7N solution in MeOH) was then added. The suspension was shaken in Parr's hydrogenator for 7 h, filtered off through diatomaceous earth pad and the filtrate was evaporated to yield **9** (230 mg, 94 %) as a dark yellow-brownish semi-solid. R_f = 0.19 ($MeOH/CH_2Cl_2$, 20:80); **^{1}H NMR** (300 MHz, $CDCl_3$): δ (ppm) 4.25-4.04 (m, 4H, OCH_2CH_3), 3.82-3.50 (overlapped m, 16H, $OC\textit{H}_2C\textit{H}2O$ and $OC\textit{H}_2CH_2N$), 3.17-3.04 (overlapped s, 2H, $NC\textit{H}_2P$), 3.04-2.71 (overlapped m, 8H $OCH_2C\textit{H}_2N$), 1.33 (t, *J* = 6.99 Hz, 6H, CH_3); **^{13}C NMR** (75 MHz, $CDCl_3$): δ (ppm) 70.4, 70.1, 69.3, 69.0, 61.9, 61.8, 55.0, 54.9, 50.2, 48.9, 48.2, 16.6, 16.5; **^{31}P NMR** (121 MHz, $CDCl_3$): δ (ppm) 25.82; **HRMS (ESI-TOF)** *m/z* $[M + Na]^+$ calcd. for $C_{17}H_{37}N_2NaO_7P^+$ 435.2231, found 435.2235.

7-Methoxy-4-(1,4,10,13-tetraoxa-7,16-diaza-cyclooctadec-7-ylmethyl)-1*H*-naphthalen-2-one (10)

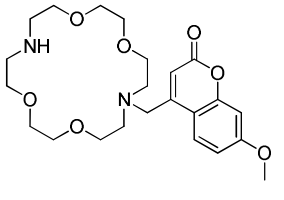

4-Bromomethyl-7-methoxycoumarin (382 mg, 1.42 mmol) was added to a stirred mixture of 4,13-diaza-18-crown 6-ether (392 g, 1.49 mmol) and $NaHCO_3$ (126 mg, 1.49 mmol) in MeCN (25 mL) and the resulting mixture was stirred at r.t. for 2 days. Then the reaction mixture was evaporated under the vacuum and the residue was purified by the column chromatography (silica gel, 3.5 % $MeOH/CH_2Cl_2$) to yield **11** (0.269 g, 40 %) as a yellowish sold: R_f = 0.39 ($MeOH/CH_2Cl_2$, 15:85); **^{1}H NMR** (300 MHz, $CDCl_3$): δ (ppm); 7.51 (d, *J* =

8.69 Hz, 1H, CC*H*C=HCC), 6.97-6.61 (overlapped m, 3H, ArC*H*), 3.86-3.77 (overlapped m, 7H, NHCH$_2$C*H*$_2$O and OC*H*$_3$), 3.67 (br s, 2H, NC*H*$_2$C), 3.59-3.51 (overlapped m, 4H, OC*H*$_2$CH$_2$O), 3.47-3.35 (overlapped m, 8H, OCH$_2$C*H*$_2$O and OC*H*$_2$CH$_2$N), 3.33-3.19 (br s, 4H, NHC*H*$_2$CH$_2$O), 2.83 (t, *J* = 8.50 Hz, OCH$_2$C*H*$_2$N); **^{13}C NMR** (75 MHz, CDCl$_3$): δ (ppm) 161.5, 160.8, 154.3, 153.0, 123.5, 111.3, 110.5, 100.1, 69.2, 68.8, 67.8, 65.4, 56.8, 54.8, 53.4, 47.1; **HRMS (ESI-TOF)** *m/z* [M + H]$^+$ calcd. for $C_{23}H_{35}N_2O_7^+$ 451.2439, found 451.2443.

General procedure for the preparation of compounds 11-16

NaHCO$_3$ (3.2 equiv.) was added to a stirred solution of the corresponding 18-crown-6 ether derivative **6**, **7**, **9**, **10** or 1-aza-18-crown-6 in dry DMF and the mixture was stirred for 45 min. The corresponding bromo derivate **2** or **5** was added at once and the reaction was stirred under the nitrogen atmosphere for three days (in case of compound **10** for five days). The reaction mixture was then filtered off and solvent was evaporated by bulb-to-bulb distillation. The obtained residue was purified on Preparative Tin-Layer Chromatography plates (PTLC) to yield the final ligands in protected forms **11-16**.

(4-{3-[2-(16-Benzyl-1,4,10,13-tetraoxa-7,16-diaza-cyclooctadec-7-yl)-acetylamino]-propyl}-7,10-bis-*tert*-butoxycarbonylmethyl-1,4,7,10tetraaza-cyclododec-1-yl)-acetic acid *tert*-butyl ester (11)

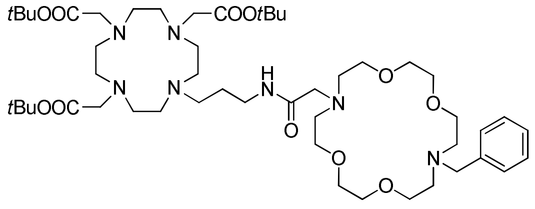

Yield: dark yellow solid (199 mg, 0.149 mmol, 72 %): R$_f$ = 0.59 (CH$_2$Cl$_2$/MeOH, 85:15); **^{1}H NMR** (300 MHz, CDCl$_3$): δ (ppm) 7.47-7.11 (overlapped m, 5H, Ar*H*), 4.13-1.98 (br, 54H, C*H*$_2$), 1.79 (br s, 2H, CH$_2$C*H*$_2$CH$_2$), 1.70-1.33 (overlapped s, 27H, C(C*H*$_3$)$_3$); **^{13}C NMR** (75 MHz, CDCl$_3$): δ (ppm): 173.5, 172.6, 172.3, 136.4, 130.4, 128.4, 127.7, 82.6, 82.4, 69.0, 68.3, 67.4, 66.9, 60.2, 57.9, 56.5, 55.8, 55.6, 53.5, 53.4, 52.2, 51.9, 50.9, 50.0, 37.7, 27.9, 27.8, 24.7; **HRMS** (ESI-TOF) *m/z* [M + Na]$^+$ calcd. for $C_{50}H_{89}N_7NaO_{11}^+$ 986.6512, found 986.6507.

(4-{3-[2-(16-Benzyl-1,4,10,13-tetraoxa-7,16-diaza-cyclooctadec-7-yl)-acetylamino]-propyl}-7-*tert*-butoxycarbonylmethyl-10-{[2-(4-nitro-phenyl)-ethylcarbamoyl]-methyl}-1,4,7,10tetraaza-cyclododec-1-yl)-acetic acid *tert*-butyl ester (12)

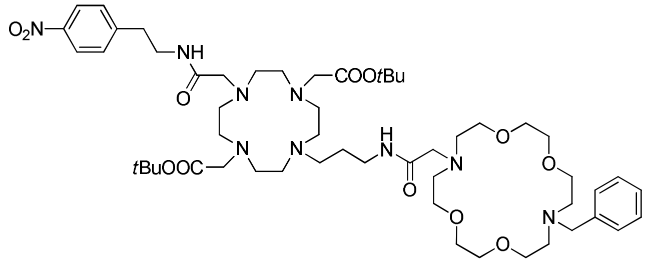

Yield: dark yellow solid (94.7 g, 79 %); R_f = 0.56 (CH_2Cl_2/MeOH, 85:15); **^{1}H NMR** (300 MHz, $CDCl_3$): δ (ppm) 8.00 (d, *J* = 8.31, 2H, Ar*H*), 7.42 (d, *J* = 8.12, 2H, Ar*H*), 7.35-6.89 (br s, 5H, Ar*H*), 3.89-1.84 (br, 58H, C*H*$_2$), 1.70 (br s, 2H, $CH_2CH_2CH_2$), 1.50-1.23 (overlapped s, 18H, C(C*H*$_3$)$_3$); **^{13}C NMR** (75 MHz, $CDCl_3$): δ (ppm) 172.8, 172.6, 171.1, 148.1, 146.3, 136.3 130.3, 130.0, 128.4, 127.8, 123.3, 82.3, 69.8, 68.8, 68.2, 67.4, 66.8, 60.3, 57.9, 57.8, 56.6, 56.2, 53.4, 52.3, 51.7, 50.2, 50.0, 48.3, 39.6, 37. 9, 35.3, 27.9, 24.3; **HRMS (ESI-TOF)** *m/z* [M + Na]$^+$ calcd. for $C_{54}H_{89}N_9NaO_{12}^+$ 1078.6523, found 1078.6520.

(16-{[3-(4,10-Bis-*tert*-butoxycarbonylmethyl-7-{[2-(4-nitro-phenyl)-ethylcarbamoyl]-methyl}-1,4,7,10tetraaza-cyclododec-1-yl)-propylcarbamoyl]-methyl}-1,4,10,13-tetraoxa-7,16-diaza-cyclooctadec-7-yl)-acetic acid *tert*-butyl ester (13)

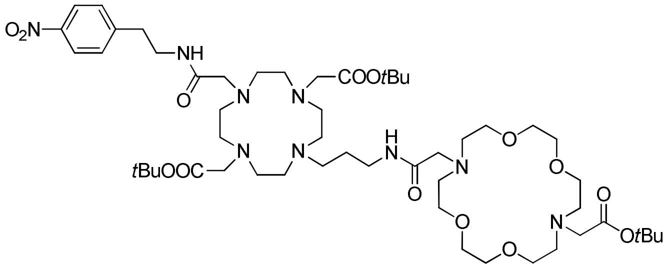

Yield: dark yellow solid (140g, 61 %); **^{1}H NMR** (300 MHz, $CDCl_3$): δ (ppm) 8.02 (d, *J* = 8.31 Hz, 2H, Ar*H*), 7.43 (d, *J* = 7.74 Hz, 2H, Ar*H*), 4.34-1.52 (br, 58H, C*H*$_2$), 1.51-1.26 (overlapped s, 27H, C(C*H*$_3$)$_3$); **^{13}C NMR** (75 MHz, $CDCl_3$): δ (ppm) 171.9, 171.3, 170.6, 170.2, 147.1, 145.4, 129.1, 122.4, 81.7, 81.4, 67.9, 67.6, 66.2, 66.0, 60.9, 58.6, 57.5, 55.8, 55.3, 53.9, 53.3, 50.9, 50.8, 49.9, 49.2, 38.7, 36.6, 34.3, 27.2, 27.2, 27.1, 24.8; **HRMS (ESI-TOF):** *m/z* [M + Na]$^+$ calcd. for $C_{53}H_{93}N_9NaO_{14}^+$ 1102.6734, found 1102.6729.

(7-*tert*-Butoxycarbonylmethyl-4-(3-{2-[16-(diethoxy-phosphorylmethyl)-1,4,10,13-tetraoxa-7,16-diaza-cyclooctadec-7-yl]-acetylamino}-propyl)-10-{[2-(4-nitro-phenyl)-ethylcarbamoyl]-methyl}-1,4,7,10tetraaza-cyclododec-1-yl)-acetic acid *tert*-butyl ester (14)

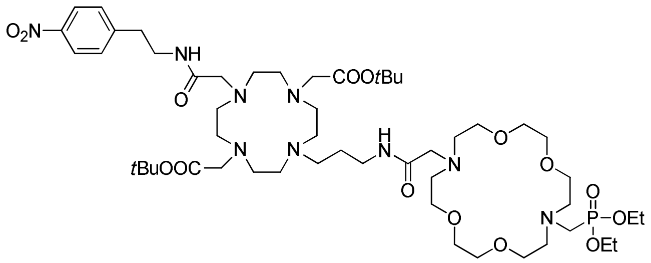

Yield: dark yellow solid (70 g, 25 %); ^{1}**H NMR** (300 MHz, $CDCl_3$): δ (ppm) 8.03 (d, J = 8.02 Hz, 2H, Ar*H*), 7.43 (d, J = 7.44 Hz, 2H, Ar*H*), 4.00 (quint, 4H, CH_2CH_3), 3.85-1.91 (br, 58H, CH_2), 1.78 (br s, 2H, $CH_2CH_2CH_2$), 1.50-1.32 (overlapped s, 18H, $C(CH_3)_3$), 1.25 (t, 6H, CH_2CH_3); ^{13}**C NMR** (75 MHz, $CDCl_3$): δ (ppm); 173.5, 172.6, 172.3, 136.4, 130.4, 128.4, 127.7, 82.6, 82.4, 69.0, 68.3, 67.4, 66.9, 60.2, 57.9, 56.5, 55.8, 55.6, 53.5, 53.4, 52.2, 51.9, 50.9, 50.0, 37.7, 32.0, 31.9, 29.6, 27.9, 27.8, 24.7; ^{31}**P NMR** (121.5 MHz, $CDCl_3$): δ (ppm) 26.50, 26.81, 27.31; **HRMS (ESI-TOF):** *m/z* $[M + Na]^+$ calcd. for $C_{52}H_{94}N_9NaO_{15}P^+$ 1138.6499, found 1138.6491.

{7-*tert*-Butoxycarbonylmethyl-4-{[2-(4-nitro-phenyl)-ethylcarbamoyl]-methyl}-10-[3-(2-1,4,7,10,13-pentaoxa-16-aza-cyclooctadec-16-yl-acetylamino)-propyl]-1,4,7,10tetraaza-cyclododec-1-yl}-acetic acid *tert*-butyl ester (15)

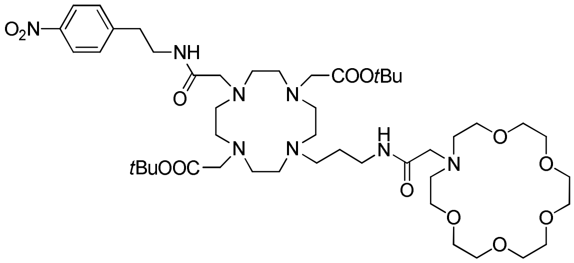

Yield: white solid (72 %). ^{1}**H NMR** (300 MHz, $CDCl_3$): δ (ppm) 8.10-7.98 (m, 2H, Ar*H*), 7.59-7.32 (m, 2H, Ar*H*), 3.92-1.91 (br, 58H, CH_2), 1.77 (broad s, 2H, $CH_2CH_2CH_2$), 1.52-1.28 (overlapped s, 18H, $C(CH_3)_3$); ^{13}**C NMR** (75 MHz, $CDCl_3$): δ (ppm) 172.9, 172.5, 171.2, 148.2, 146.4, 130.1, 123.3, 82.3, 81.7, 68.9, 66.6, 68.5, 68.1, 67.1, 58.2, 56.3, 54.4, 54.1, 53.5, 51.8, 50.3, 50.0, 48.9, 39.6, 37.9, 35.3, 28.0, 24.1; **HRMS (ESI-TOF):** *m/z* $[M + Na]^+$ calcd. for $C_{47}H_{82}N_8NaO_{13}^+$ 989.5894, found 989.5887.

Carboxymethyl-4-(3-{2-[16-(6-methoxy-3-oxo-3,4-dihydro-naphthalen-1-ylmethyl)-1,4,10,13-tetraoxa-7,16-diaza-cyclooctadec-7-yl]-acetylamino}-propyl)-7-*tert*-butoxycarbonylmethyl-10-{[2-(4-nitro-phenyl)-ethylcarbamoyl]-methyl}-1,4,7,10tetraaza-cyclododec-1-yl)-acetic acid *tert*-butyl ester (16)

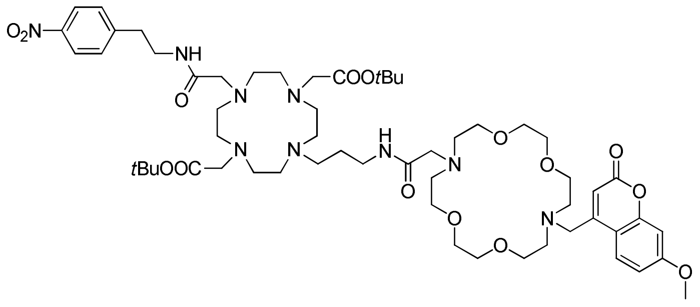

Yield: white solid (80 mg, 26 %). **^{1}H NMR** (300 MHz, $CDCl_3$): δ (ppm) 8.19-7.87 (m, 2H, Ar*H*), 7.62 (dd, *J* = 59.0, 8.78 Hz, 1H, Ar*H*), 7.48-7.33 (m, 2H, Ar*H*), 7.03-6.59 (overlapped m, 2H, Ar*H*), 6.44 (d, *J* = 25.12 Hz, 1H, C=C*H*CO_2C), 4.07-2.00 (br, 61H, CH_2 and CH_3), 1.83-1.60 (br, $CH_2CH_2CH_2$), 1.51-1.26 (overlapped s, 18H, $C(CH_3)_3$); **^{13}C NMR** (75 MHz, $CDCl_3$): δ (ppm); 171.4, 170.2, 168.9, 161.7, 161.5, 160.2, 154.3, 151.9, 147.2, 146.6, 145.5, 129.0, 123.8, 122.5, 111.9, 111.5, 110.2, 100.1, 80.8, 67.9, 67.9, 67.1, 66.1, 57.8, 57.1, 55.3, 55.0, 53.6, 53.4, 53.0, 52.4, 50.7, 50.2, 49.4, 48.4, 46.0, 38.6, 36.9, 34.3, 28.7, 27.2, 27.0, 22.4; **HRMS (ESI-TOF):** *m/z* $[M + Na]^+$ calcd. for $C_{58}H_{91}N_9NaO_{15}^+$ 1176.6527, found 1176.6523.

General hydrolysis procedure of compounds 11-13, 15 and 16 to obtain $L^{1\text{-}3}$, L^5 and L^6

The compound **11-13**, **15** or **16** was dissolved in formic acid (8 mL) and stirred at 60 °C for 24 hours. After the hydrolysis completion was confirmed by the low resolution MS analysis, the reaction was stopped and the solvent was evaporated under the reduced pressure followed by lyophilization for two days to yield the corresponding final ligand $\mathbf{L^{1\text{-}3,5,6}}$. The products were analyzed by CHN analysis and the amount of ligand was calculated with respect to the content of nitrogen for the subsequent complexation with Ln^{3+}.

(4-{3-[2-(16-Benzyl-1,4,10,13-tetraoxa-7,16-diaza-cyclooctadec-7-yl)-acetylamino]-propyl}-7,10-bis-carboxymethyl-1,4,7,10tetraaza-cyclododec-1-yl)-acetic (L^1)

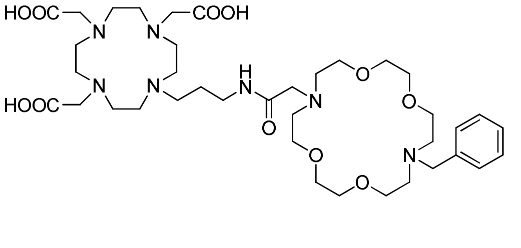

Yield: off-white solid (75 %). **^{1}H NMR** (300 MHz, D_2O): δ (ppm) 7.50 (br s, 5H, Ar*H*), 4.48 (br s, 2H, CH_2Ph), 4.16 (br s, 2H, $CH_2C(O)NH$), 4.00-2.52 (br, 50H, CH_2), 1.76 (br s, 2H, $CH_2CH_2CH_2$); **^{13}C NMR** (75 MHz, D_2O): δ (ppm): 171.7, 171.1, 164.0, 130.5, 129.5, 128.5, 127.6, 69.0, 68.8, 63.0, 62.5, 57.7, 54.3, 53.7, 53.5, 52.1, 51.9, 50.0, 49.0, 48.6, 48.2, 36.3, 23.3; **HRMS (ESI-TOF)** *m/z* $[M + 2H]^{2+}$ calcd. for $C_{38}H_{67}N_7O_{11}{}^{2+}$ 398.7444, found 398.7448.

(4-{3-[2-(16-Benzyl-1,4,10,13-tetraoxa-7,16-diaza-cyclooctadec-7-yl)-acetylamino]-propyl}-7-carboxymethyl-10-{[2-(4-nitro-phenyl)-ethylcarbamoyl]-methyl}-1,4,7,10tetraaza-cyclododec-1-yl)-acetic acid (L^2)

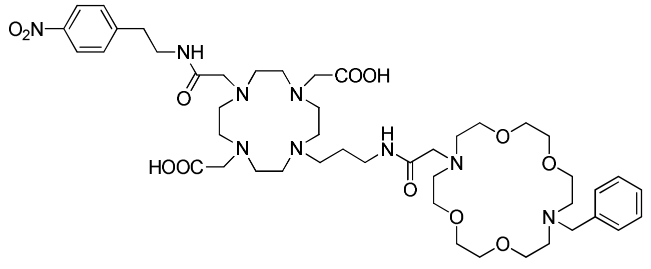

Yield: off-white solid (72 %). **^{1}H NMR** (300 MHz, D_2O): δ (ppm) 8.02 (d, J = 8.50 Hz, 2H, Ar*H*), 7.46-7.23 (overlapped m, 5H, Ar*H*), 4.35 (br s, 2H, $CH_2CONH(CH_2)_2$), 4.03 (br s, 2H, $CH_2CONH(CH_2)_3$), 3.82-2.71 (overlapped m, 52H, CH_2), 2.62 (br s, 2H, $CH_2(CH_2)_2NHCO$), 1.63 (quint, 2H, $CH_2CH_2CH_2$); **^{13}C NMR** (75 MHz, D_2O): δ (ppm) 171.2, 169.0, 164.8, 147.9, 147.3, 146.3, 131.4, 130.4, 130.1, 129.4, 123.6, 69.9, 69.7, 63.9, 63.4, 58.6, 56.7, 56.4, 55.8, 54.6, 54.4, 53.0, 50.6, 50.1, 49.1, 48.5, 39.7, 37.4, 34.7, 24.5; **HRMS (ESI-TOF)** *m/z* $[M + 2H]^{2+}$ calcd. for $C_{46}H_{75}N_9O_{12}{}^{2+}$ 472.7762, found 472.7771.

(16-{[3-(4,10-Bis-carboxymethyl-7-{[2-(4-nitro-phenyl)-ethylcarbamoyl]-methyl}-1,4,7,10tetraaza-cyclododec-1-yl)-propylcarbamoyl]-methyl}-1,4,10,13-tetraoxa-7,16-diaza-cyclooctadec-7-yl)-acetic acid (L^3)

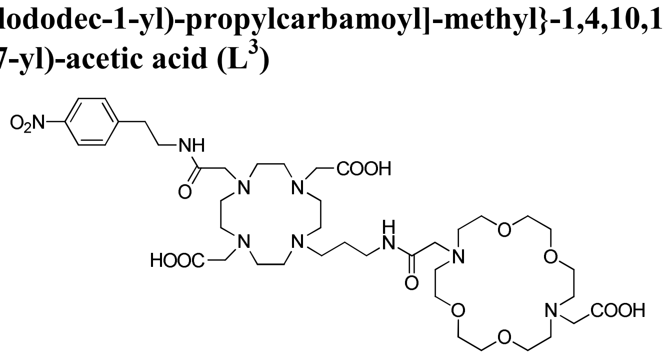

Yield: off-white solid (82 %). **^{1}H NMR** (300 MHz, D_2O): δ (ppm) 8.02 (d, J = 8.12 Hz, 2H, Ar*H*), 7.34 (d, J = 7.93 Hz, 2H, Ar*H*), 4.01-1.99 (overlapped m, 58H, CH_2), 1.57 (br s, 2H,

$CH_2CH_2CH_2$); **^{13}C NMR** (75 MHz, D_2O): δ (ppm) 169.9, 168.7, 168.4, 168.3, 146.6, 145.0, 128.8, 122.3, 68.6, 68.3, 65.8, 62.5, 55.6, 53.8, 53.5, 53.0, 50.1, 49.8, 48.7, 48.6. 46.6, 46.5, 46.3; 38.2, 36.0, 33.4, 23.2; **HRMS (ESI-TOF):** *m/z* $[M + 2H]^{2+}$ calcd. for $C_{41}H_{71}N_9O_{14}^{2+}$ 456.77621, found 456.7755.

(7-Carboxymethyl-4-{[2-(4-nitro-phenyl)-ethylcarbamoyl]-methyl}-10-{3-[2-(16-phosphonomethyl-1,4,10,13-tetraoxa-7,16-diaza-cyclooctadec-7-yl)-acetylamino]-propyl}-1,4,7,10tetraaza-cyclododec-1-yl)-acetic acid (L^4)

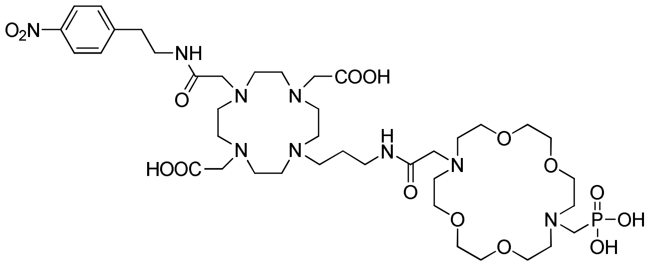

The compound **14** (70 mg, 63 mmol) was dissolved in dry CH_2Cl_2 (5 mL) in a flame dried flask, and the solution was cooled down to °0 C under the nitrogen atmosphere. Then the $BrSiMe_3$ (196 mg, 1.254 mmol) was added slowly and the reaction was stirred for 2 h at r.t., to result in formation of a white precipitate. The solvent was then evaporated under the reduced pressure, H_2O (3 mL) was added without previous isolation into the solid residue and the mixture was stirred for 1.5 h. The reaction mixture was then evaporated and lyophilized to give glassy amorphous solid (32 mg, 54 %). **^{1}H NMR** (300 MHz, D_2O): δ (ppm) 8.04 (d, *J* = 8.50, 2H, Ar*H*), 7.36 (d, *J* = 8.31, 2H, Ar*H*), 4.07 (br s, 2H, $CH_2CONH(CH_2)_2$), 3.97 (br s, 2H, $CH_2CONH(CH_2)_3$), 3.84-2.78 (br, 54H, CH_2), 1.91 (quint. 2H, $CH_2CH_2CH_2$); **^{13}C NMR** (75 MHz, D_2O): δ (ppm) 174.0, 165.2, 164.5, 147.8, 146.3, 130.2, 123.7, 69.9, 63.8, 59.8, 57.5, 55.3, 55.2, 54.7, 54.7, 54.6, 53.0, 52.2, 51.9, 51.8, 50.1, 50.1, 48.2, 48.2, 40.2, 36.9, 34.9, 22.9; **^{31}P NMR** (121.5 MHz, D_2O): δ (ppm) 6.63, 9.00; **HRMS (ESI-TOF)** *m/z* $[M + Ca]^{2+}$ calcd. for $C_{40}H_{70}CaN_9O_{15}P^{2+}$ 493.7172, found 493.7167.

{7-Carboxymethyl-4-{[2-(4-nitro-phenyl)-ethylcarbamoyl]-methyl}-10-[3-(2-1,4,7,10,13-pentaoxa-16-aza-cyclooctadec-16-yl-acetylamino)-propyl]-1,4,7,10tetraaza-cyclododec-1-yl}-acetic acid (L[5])

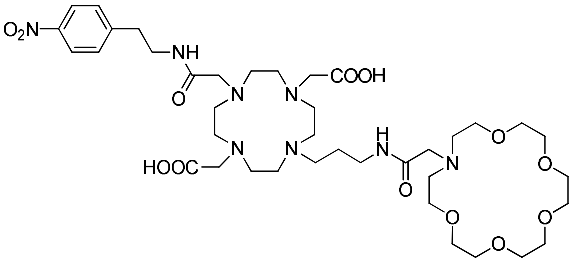

Yield: off-white solid (72 %); **^{1}H NMR** (300 MHz, D_2O): δ (ppm) 8.14 (d, *J* = 8.12 Hz, 2H, Ar*H*), 7.45 (d, *J* = 7.74 Hz, 2H, Ar*H*), 4.05 (br s, 2H, C*H*$_2$CONH), 3.93-2.75 (overlapped m, 52H, C*H*$_2$), 2.67 (br s, 2H, C*H*$_2$(CH_2)$_2$NHCO), 1.73 (quint, 2H, CH_2C*H*$_2$$CH_2$); **^{13}C NMR** (75 MHz, D_2O): δ (ppm) 170.6, 165.1, 164.9, 147.9, 146.3, 130.1, 123.7, 69.9, 69.7, 69.5, 65.9, 63.8, 56.3, 55.1, 54.6, 51.4, 51.0, 50.6, 50.3, 48.7, 48.3, 47.9, 39.7, 37.3, 34.7, 24.5; **HRMS (ESI-TOF):** *m/z* $[M + 2H]^{2+}$ calcd. for $C_{39}H_{68}N_8O_{13}{}^{2+}$ 428.2447, found 428.2450.

(7-Carboxymethyl-4-(3-{2-[16-(6-methoxy-3-oxo-3,4-dihydro-naphthalen-1-ylmethyl)-1,4,10,13-tetraoxa-7,16-diaza-cyclooctadec-7-yl]-acetylamino}-propyl)-10-{[2-(4-nitro-phenyl)-ethylcarbamoyl]-methyl}-1,4,7,10tetraaza-cyclododec-1-yl)-acetic acid (L[6])

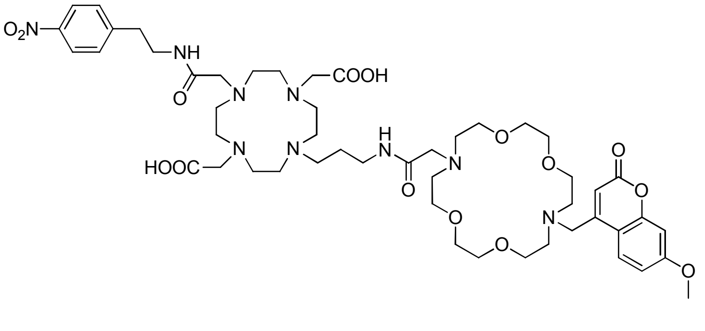

Yield: off-white solid (69 %); **^{1}H NMR** (300 MHz, D_2O): δ (ppm) 7.99 (d, *J* = 8.31 Hz, 2H, Ar*H*), 7.62 (d, *J* = 9.06 Hz, 1H, Ar*H*), 7.36 (d, *J* = 8.50 Hz, 2H, Ar*H*), 6.95 (dd, *J* = 8.88, 2.27 Hz, 1H, Ar*H*), 6.89-6.77 (m, 1H, C=C*H*COMe), 6.61 (br s, 1H, C=C*H*CO_2C), 4.09 (br s, 2H, NC*H*$_2$C=CH), 3.95-2.73 (br, 57H, C*H*$_2$ and OC*H*$_3$), 2.51 (br s, 2H, NC*H*$_2$$CH_2$$CH_2$), 1.63 (br s, 2H, CH_2C*H*$_2$$CH_2$); **^{13}C NMR** (75 MHz, D_2O): δ (ppm) 170.2, 169.5, 164.7, 163.3, 162.5, 154.9, 147.9, 146.1, 145.8, 130.0, 125.5, 123.5, 114.0, 113.4, 111.0, 101.5, 69.9, 69.8, 64.1, 63.3, 56.4, 56.2, 56.1, 55.5, 54.6, 54.4, 53.6, 51.2, 50.7, 50.0, 48.4, 48.1, 39.7, 37.5, 34.7, 24.8; **HRMS (ESI-TOF):** *m/z* $[M + 2H]^{2+}$ calcd. for $C_{50}H_{77}N_9O_{15}{}^{2+}$ 521.7764, found 521.7766.

General synthetic procedure for the metalation of ligands L^{1-6}

The final ligand $\mathbf{L^{1-6}}$ was dissolved in MilliQ H_2O (3 mL) and pH of the reaction media was adjusted to 7.2 with aqueous solution of NaOH (0.2 M). Then the corresponding amount of $LnCl_3$ dissolved in MilliQ H_2O (1 mL) was added in a dropwise manner over 0.5 h during which the pH of reaction was maintained at 7.2. After the addition was completed the temperature of the reaction mixture was elevated at 60 °C and stirred overnight. Then the solvent was evaporated under the reduced pressure and the acquired complex was freeze-dried on lyophilizer for 24 h.

$\mathbf{GdL^1}$: White solid. LRMS *m/z* $[M+H]^+$ calcd. for $C_{38}H_{63}GdN_7O_{11}^+$ 951.4, found 951.4.

$\mathbf{GdL^2}$**:** White solid. LRMS *m/z* $[M]^+$ calcd. for $C_{46}H_{71}GdN_9O_{12}^+$ 1099.4, found 1099.5.

$\mathbf{GdL^3}$**:** White solid. LRMS *m/z* $[M + Na]^+$ calcd. for $C_{41}H_{66}GdN_9NaO_{14}^+$ 1089.4, found 1089.4.

$\mathbf{EuL^3}$**:** White solid. LRMS *m/z* $[M + Na]^+$ calcd. for $C_{41}H_{66}EuN_9NaO_{14}^+$ 1084.4, found 1084.4.

$\mathbf{GdL^4}$**:** White solid. LRMS *m/z* $[M + Na]^+$ calcd. for $C_{40}H_{67}GdN_9NaO_{15}P^+$ 1125.4, found 1125.4.

$\mathbf{GdL^5}$**:** Off-white solid. LRMS *m/z* $[M + H]^{2+}$ calcd. for $C_{39}H_{65}GdN_8O_{13}^{2+}$ 505.7, found 505.7.

$\mathbf{EuL^5}$**:** Off-white solid. LRMS *m/z* $[M + H]^{2+}$ calcd. for $C_{39}H_{65}EuN_8O_{13}^{2+}$ 503.2, found 503.3.

$\mathbf{GdL^6}$**:** Dark yellow solid. LRMS *m/z* $[M + H]^{2+}$ calcd. for $C_{50}H_{74}GdN_9O_{15}^{2+}$ 599.2, found 599.3.

Purification of the ligands

Solvents for the preparative RP HPLC purification were buffered with 0.1 % HCOOH and the compounds were eluted using the method given in Table S1, with the flow rate of 10 mL/min.

Table S1. Method used in RP HPLC purification.

Time (min.)	0:0	1:00	3:00	5:00	7:00	15:00	25:00	30:00
MeCN (%)	0	5	10	15	20	25	50	90
H_2O (%)	100	95	90	85	80	75	50	10

2. Relaxometric studies

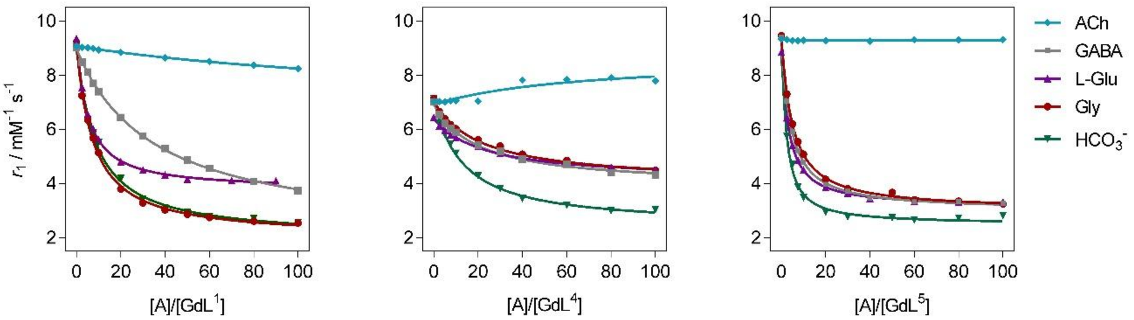

Figure S1. ^{1}H longitudinal relaxometric titration profiles for **GdL^{1,4,5}** with the different analytes ([**GdL^{1,4,5}**] = 3.0 mM, 298 K, pH 7.4, 50 mM HEPES, at 7.05 T).

Table S2. Binding affinity constants and relaxivity decrease for complexes **GdL$^{1-6}$** upon addition of 60 equiv. of analytes.[a]

Affinity const. $K_{GdL^{1-6}}$ (M^{-1})	Gly	GABA	L-Glu	HCO_3^-	ACh
K_{GdL^1}	47	10	73	40	2
K_{GdL^2}	68	78	311	216	N/A
K_{GdL^3}	23	19	26	72	N/A
K_{GdL^4}	16	17	15	26	5
K_{GdL^5}	76	92	114	188	N/A
K_{GdL^6}	69	20	19	150	1

[a] *K*a were obtained by fitting ^{1}H NMR relaxometric titrations curves according to Equation 2.

3. Luminescence studies

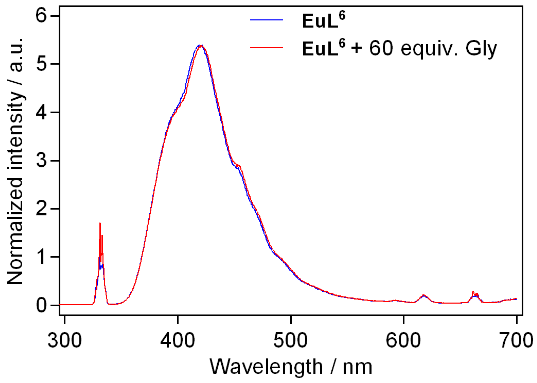

Figure S2. Luminescence emission spectra of **EuL**^{6} before and after the addition 50 equiv. of Gly (λ_{ex} = 330 nm, [**EuL**^{6}] = 50 µM, pH 7.4, 50 mM HEPES, 298 K).

Table S3. Ratio of $^5D_0 - {}^7F_2$ to $^5D_0 - {}^7F_1$ for **EuL**^{3,5} before and after addition 50 equiv. of Gly (λ_{ex} = 395 nm, [**EuL**^{3,5}] = 5 mM, pH 7.4 in 50 mM HEPES, 298 K).

Complex	EuL^3	EuL^3-Gly	EuL^5	EuL^5-Gly
**Ratio**	3.17	3.20	1.80	3.37

4. NMR Spectra of ligands L^{1-6}

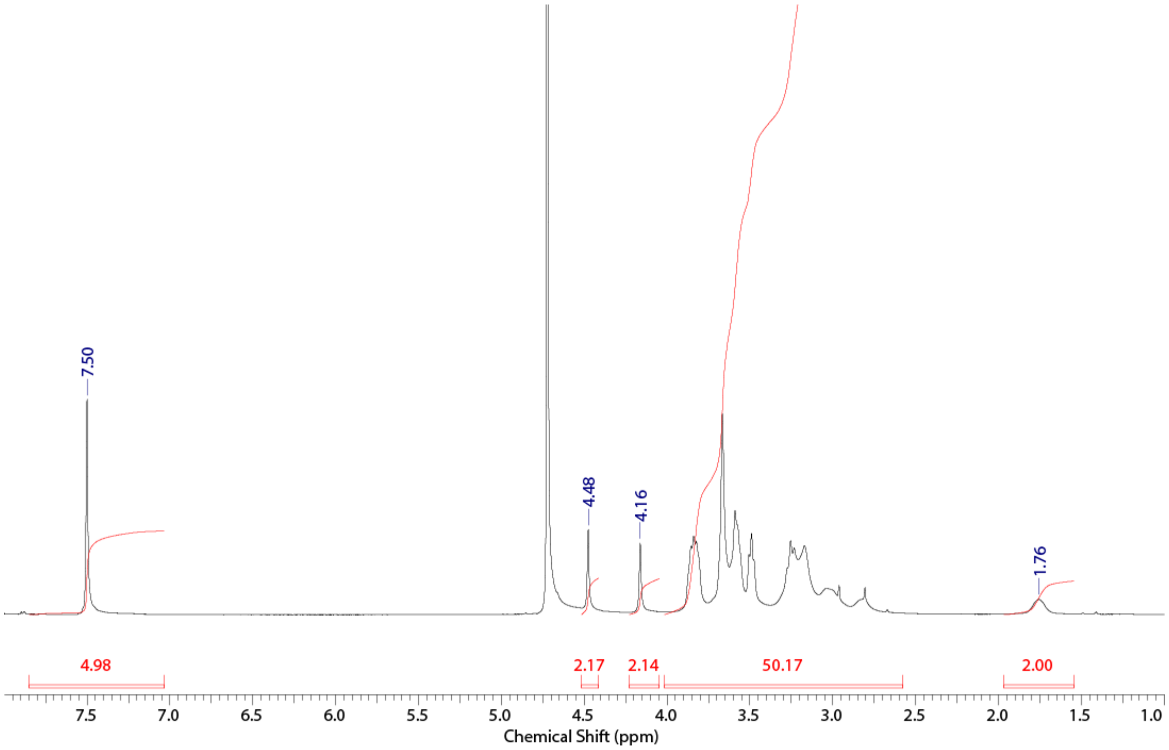

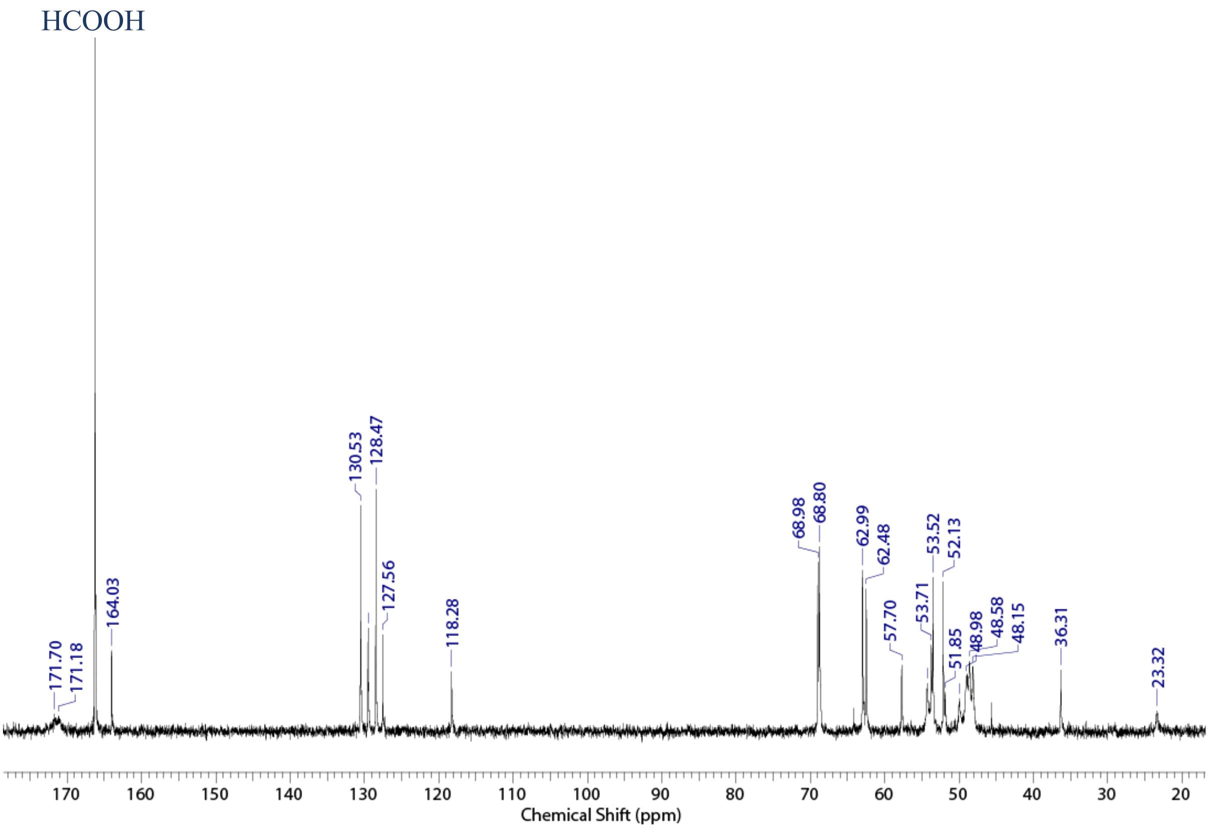

Figure S3. ^{1}H (300 MHz, top) and ^{13}C (75 MHz, bottom) NMR spectra of $\mathbf{L^1}$ in D_2O (298 K)

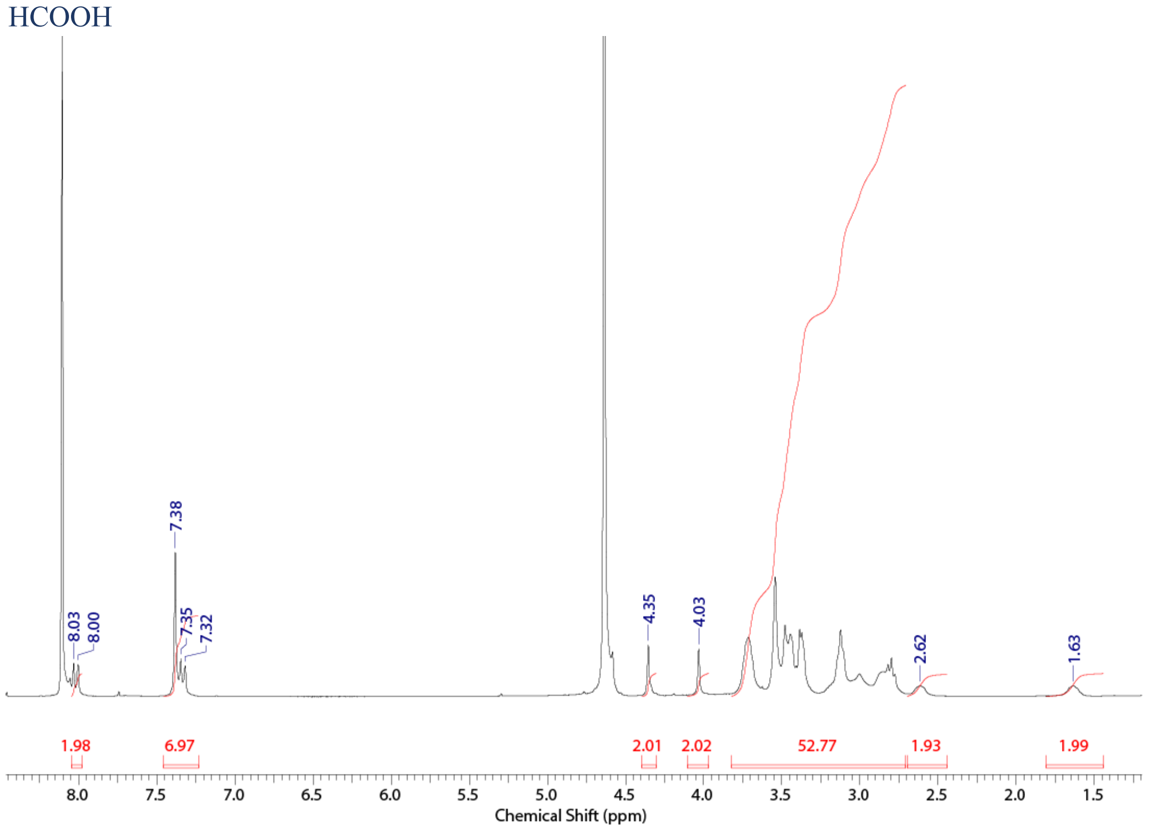

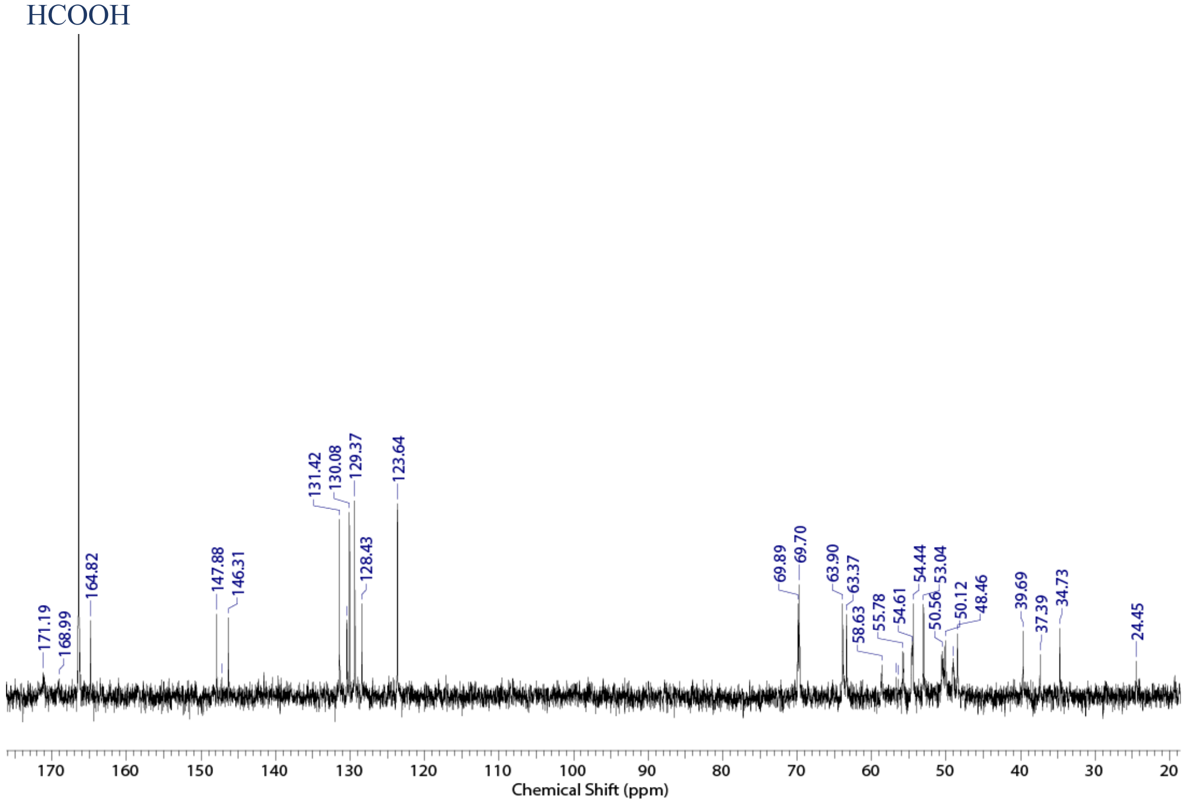

Figure S4. ^{1}H (300 MHz, top) and ^{13}C (75 MHz, bottom) NMR spectra of $\mathbf{L^2}$ in D_2O (298 K)

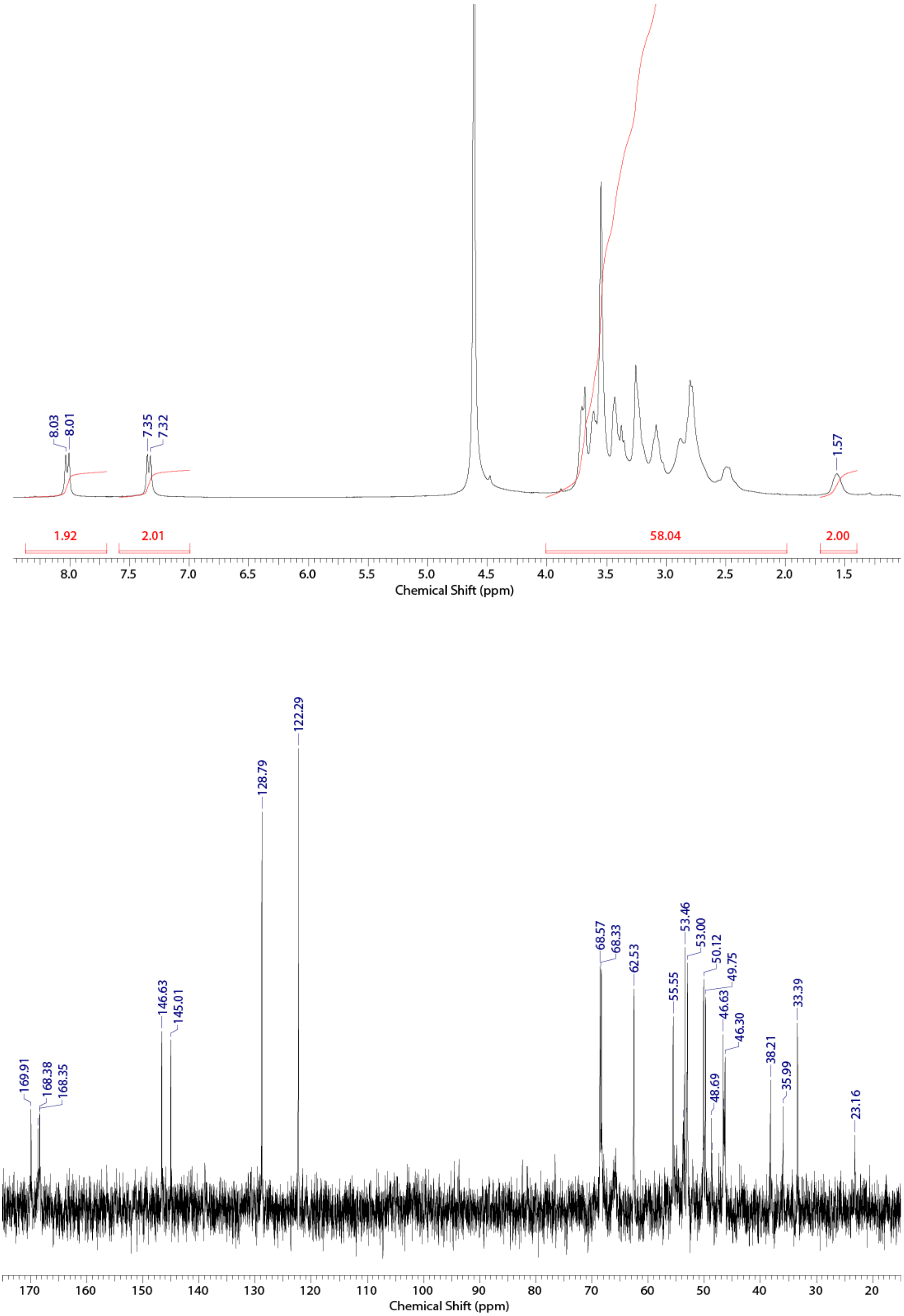

Figure S5. ^{1}H (300 MHz, top) and ^{13}C (75 MHz, bottom) NMR spectra of **L^{3}** in D_2O (298 K)

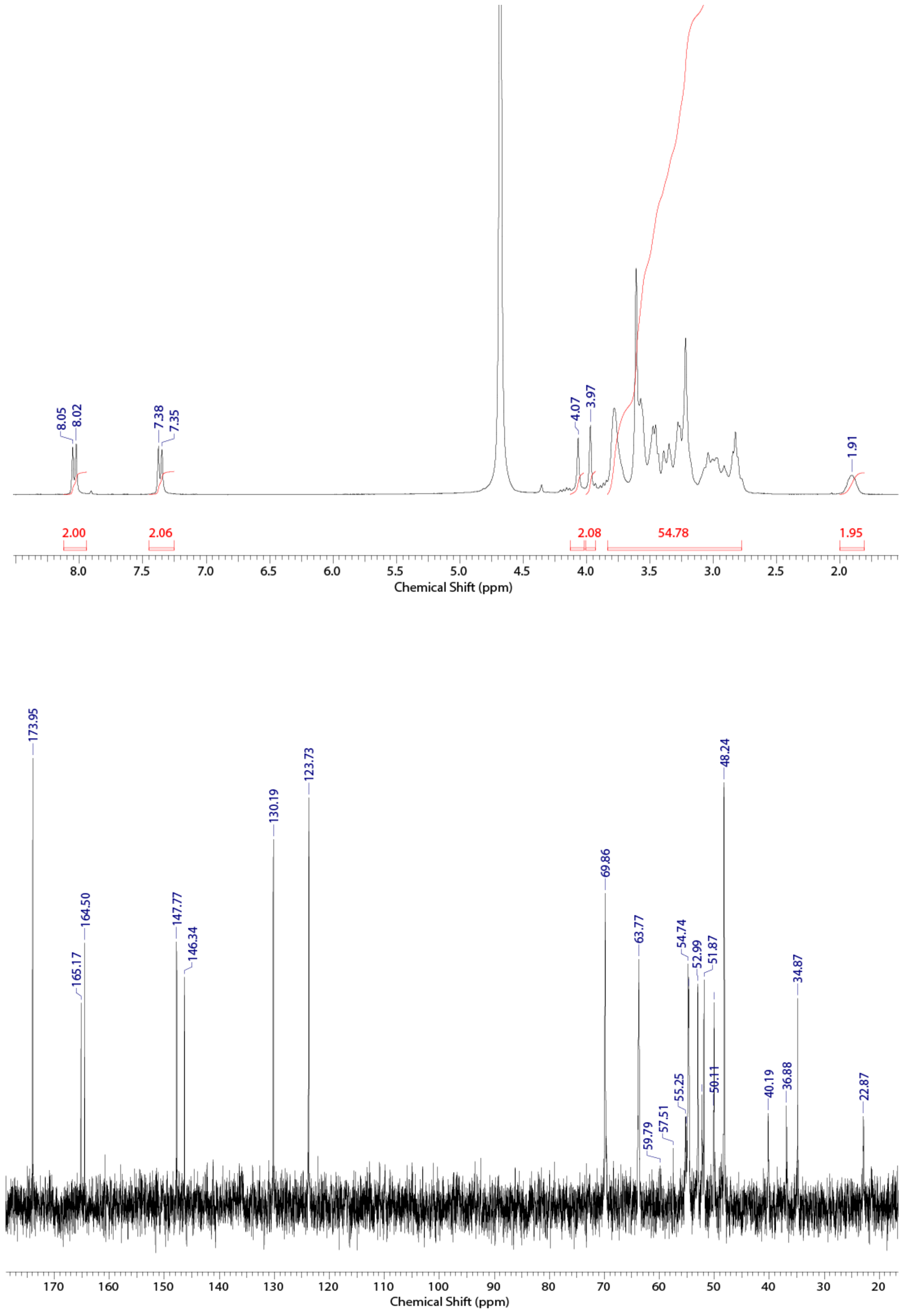

Figure S6. ^{1}H (300 MHz, top) and ^{13}C (75 MHz, bottom) NMR spectra of $\mathbf{L^4}$ in D_2O (298 K)

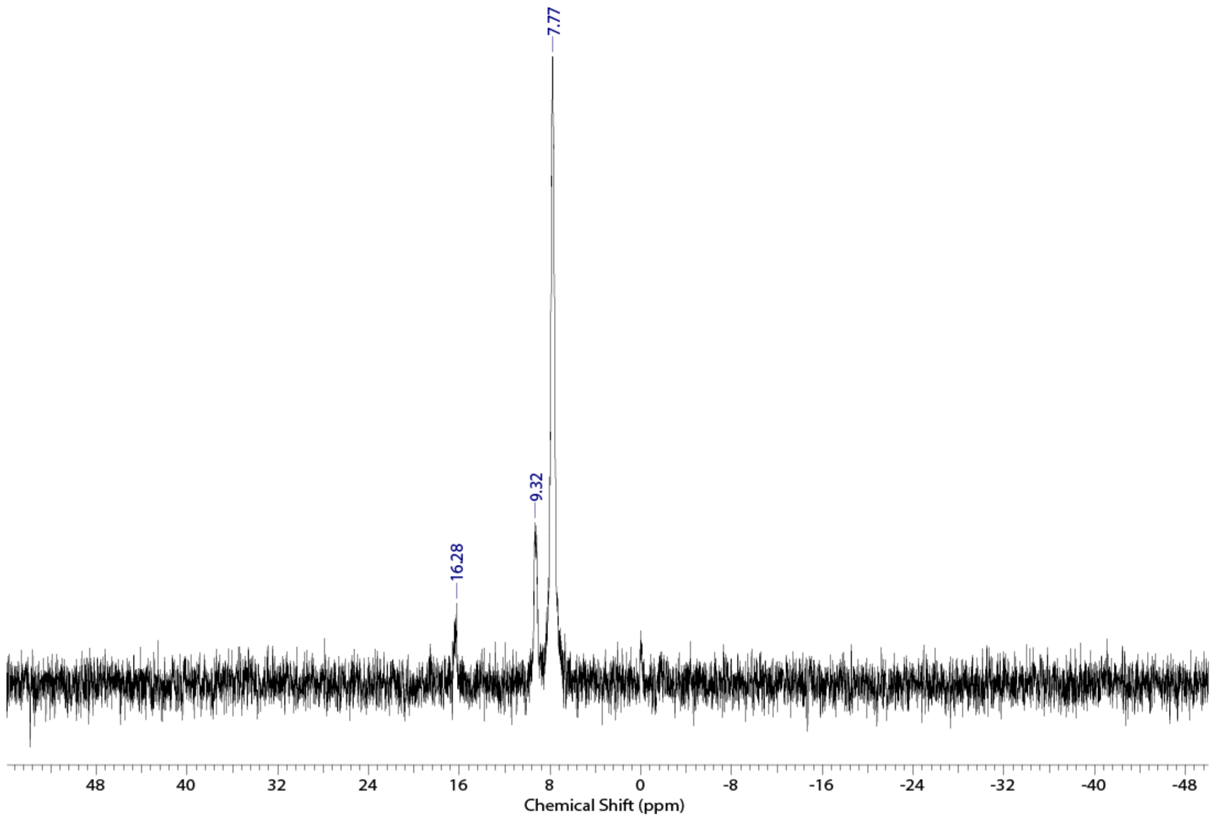

Figure S7. ^{31}P NMR spectra of $\mathbf{L^4}$ in D_2O (121 MHz, 298 K)

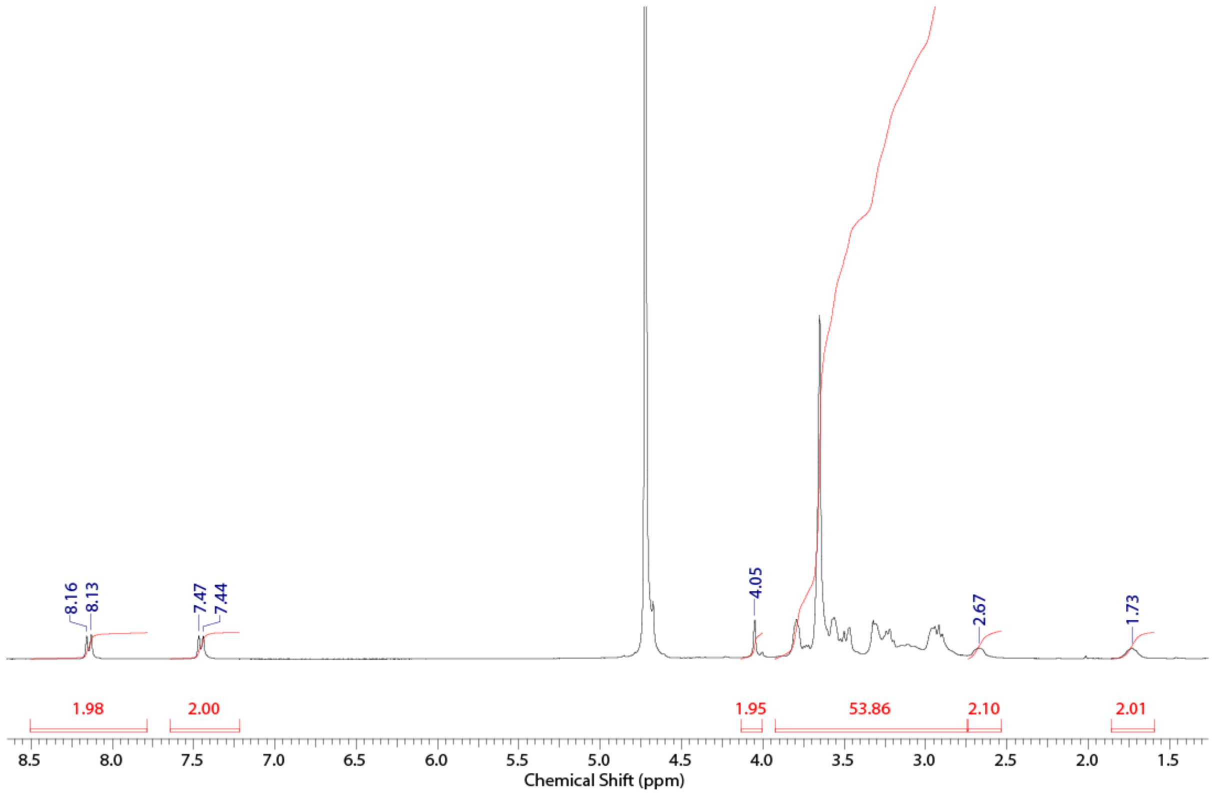

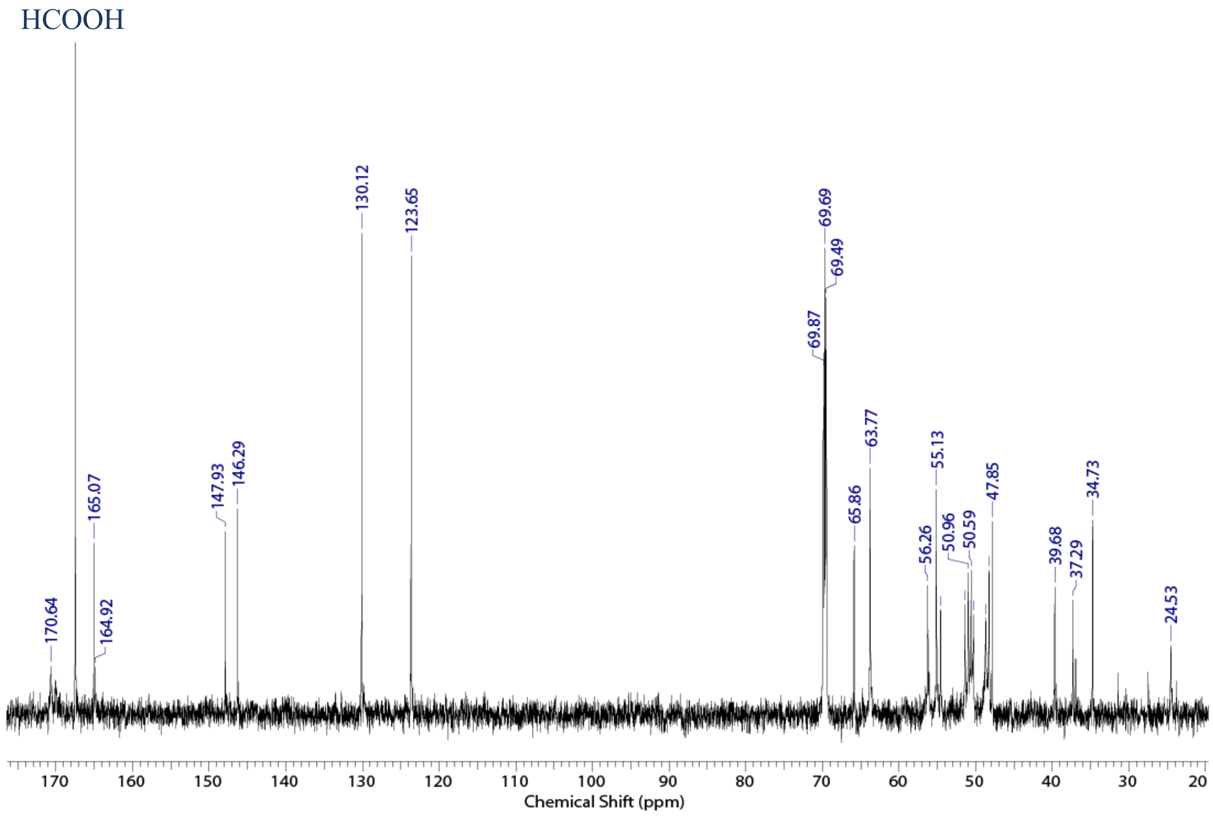

Figure S8. ^{1}H (300 MHz, top) and ^{13}C (75 MHz, botom) NMR spectra of $\mathbf{L^5}$ in D_2O (298 K)

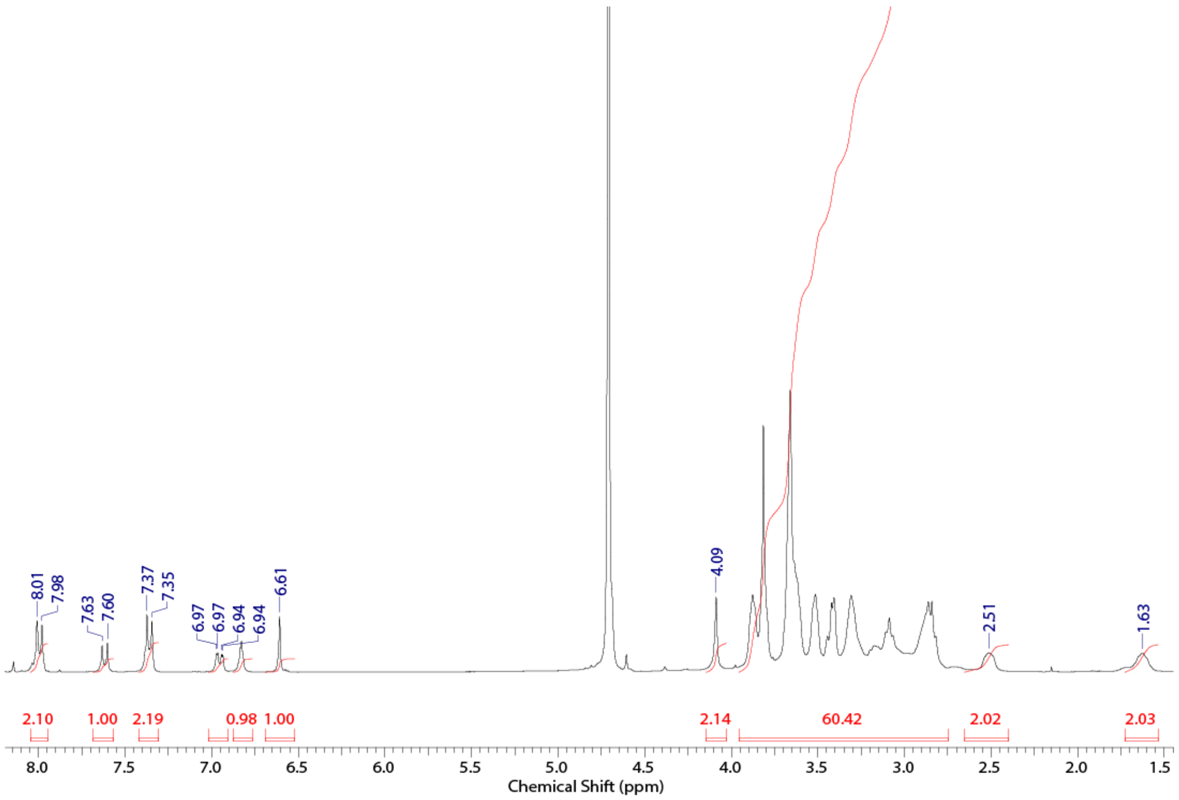

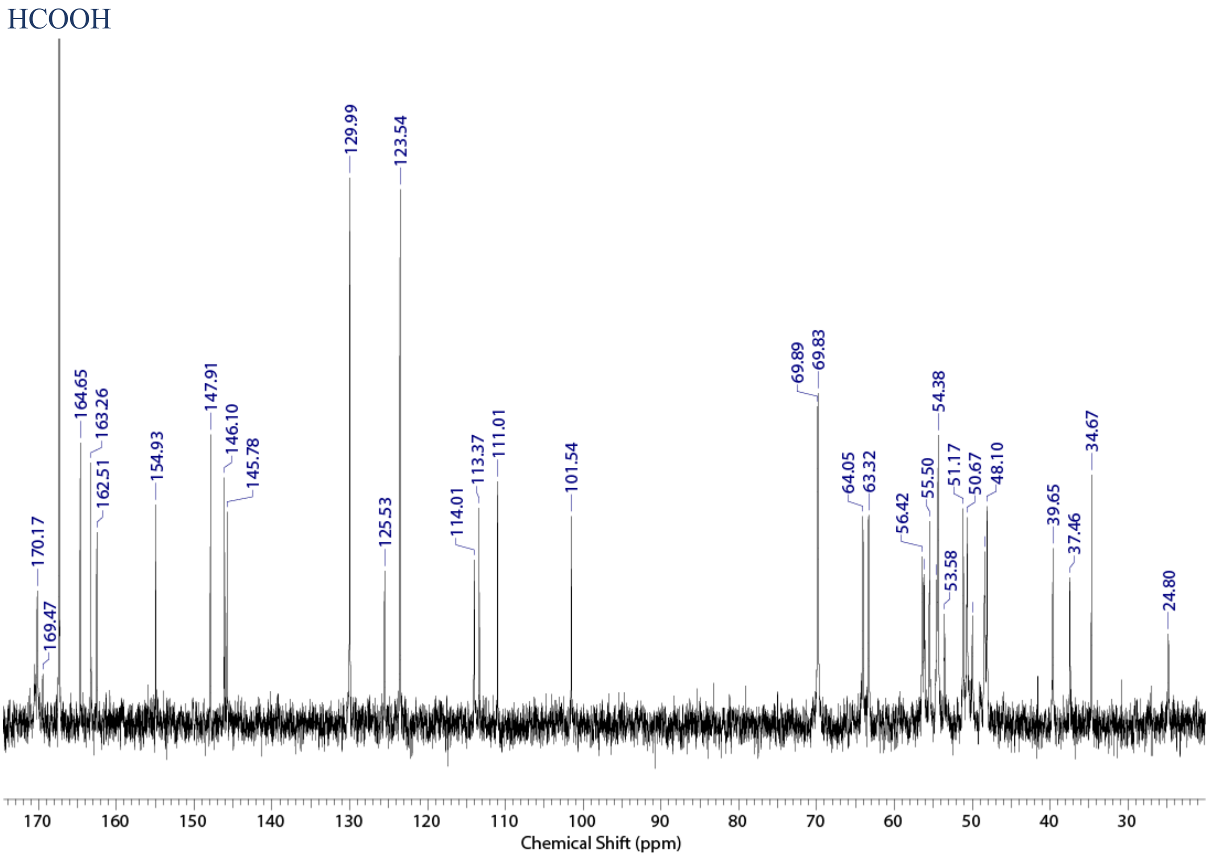

Figure S9. ^{1}H (300 MHz, top) and ^{13}C (75 MHz, botom) NMR spectra of **L**^{6} in D_2O (298 K)

5. DFT calculations

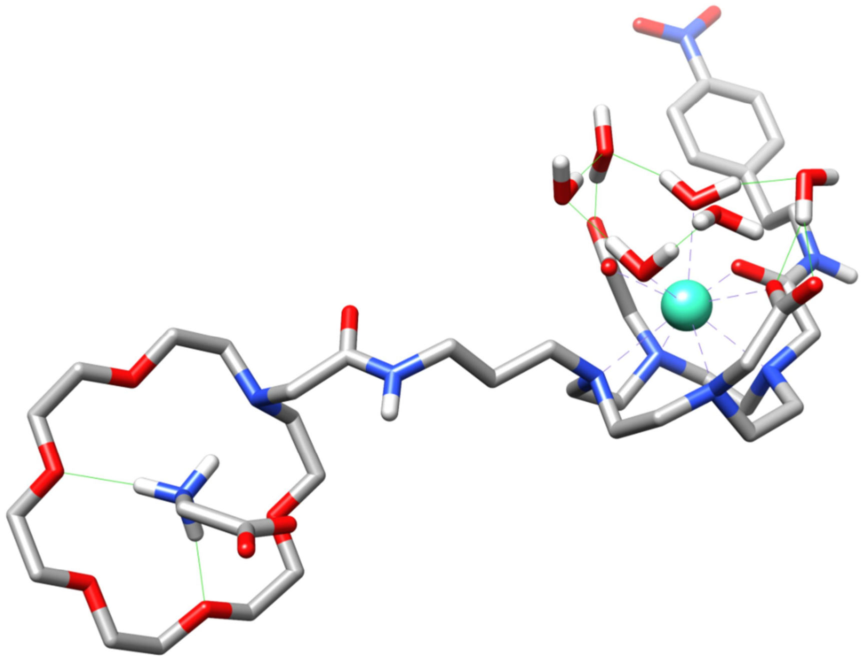

Figure S10. Structure of the **GdL**^{5}(Gly)(H_2O)$_2$·4H_2O system obtained with DFT calculations showing the binding of Gly to the crown moiety.

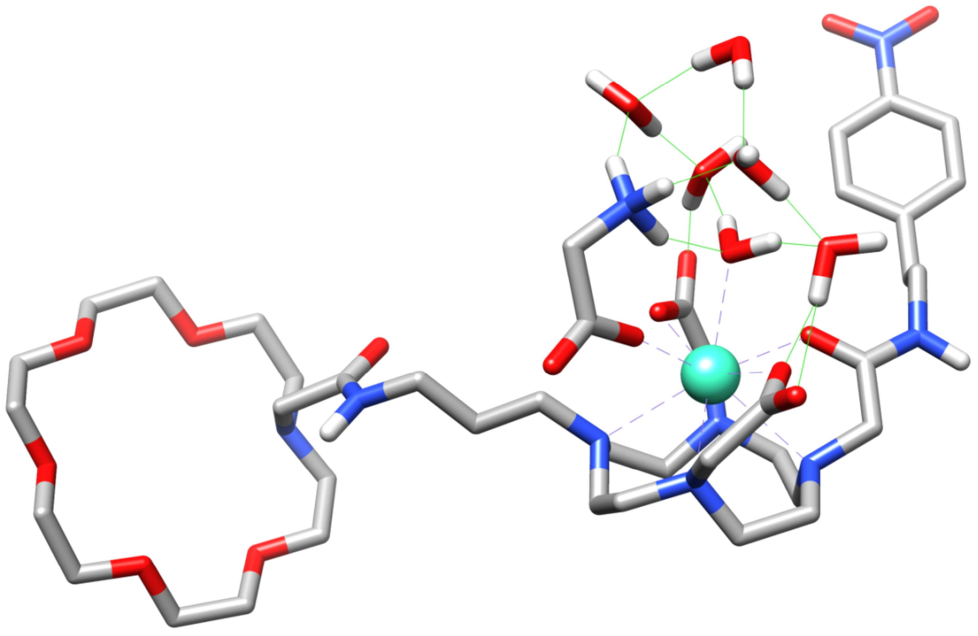

Figure S11. Structure of the **GdL**^{5}(Gly)(H_2O)·5H_2O system obtained with DFT calculations showing the binding of Gly to the metal ion.

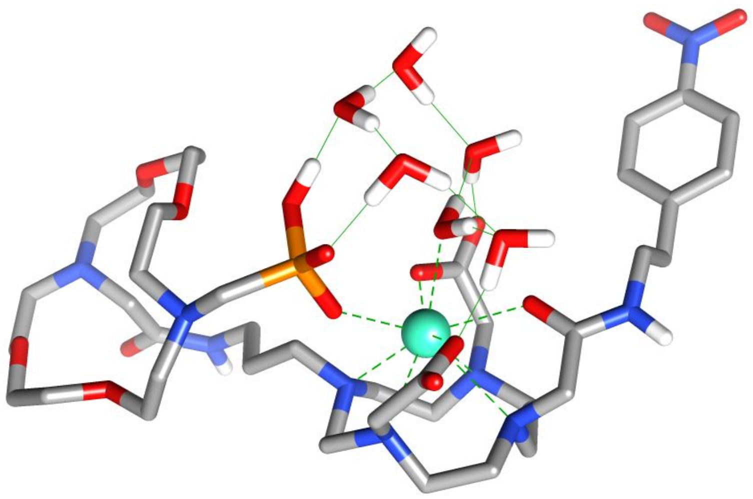

Figure S12. Structure of the **GdL[4]**(H_2O)·5H_2O system obtained with DFT calculations showing the coordination of the remote phosphonate group to the metal ion.

Optimized Cartesian coordinates (Å) obtained for the GdL^3(H_2O)$_2$·4H_2O system with DFT calculations.

Center Number	Atomic Number	X	Y	Z
1	7	3.656681	-0.486854	-2.271661
2	6	4.546563	-1.405997	-3.036015
3	6	4.236703	-2.877518	-2.771574
4	7	4.345357	-3.270509	-1.337541
5	6	3.608066	-4.547891	-1.108625
6	6	2.101060	-4.334080	-1.030944
7	7	1.682117	-3.446723	0.089528
8	6	0.282287	-2.975572	-0.137402
9	6	0.177832	-1.916576	-1.233286
10	7	0.913848	-0.672182	-0.886058
11	6	1.214802	0.140938	-2.095757
12	6	2.334781	-0.430306	-2.953327
13	6	4.266077	0.869652	-2.173423
14	6	4.074309	1.528336	-0.801536
15	8	3.254010	0.964956	0.008405
16	8	4.729019	2.562297	-0.535719
17	6	5.761032	-3.445110	-0.949568
18	6	6.402572	-2.107902	-0.621256
19	8	5.692417	-1.156068	-0.213412
20	6	1.765942	-4.175730	1.380745
21	6	3.108384	-4.053720	2.112741
22	8	3.247091	-4.691522	3.172726
23	8	3.981209	-3.235527	1.620171
24	1	5.340766	0.795414	-2.347589
25	1	3.867377	1.540466	-2.946845
26	1	6.333639	-3.990879	-1.711976
27	1	5.788390	-4.026721	-0.022993
28	1	1.518352	-5.238735	1.261140
29	1	1.025083	-3.752981	2.065305
30	1	5.579246	-1.183977	-2.765677
31	1	4.458322	-1.227129	-4.117592
32	1	4.910510	-3.493502	-3.384895
33	1	3.221119	-3.106419	-3.100324
34	1	3.821154	-5.268658	-1.910409
35	1	3.974121	-4.978906	-0.176066
36	1	1.611796	-5.314483	-0.935730
37	1	1.741044	-3.895297	-1.963294
38	1	-0.373159	-3.821036	-0.391006
39	1	-0.067838	-2.555633	0.808030
40	1	0.575575	-2.300584	-2.175472
41	1	-0.885967	-1.713842	-1.414607
42	1	1.486037	1.139716	-1.745694
43	1	0.324190	0.241627	-2.731911
44	1	2.077005	-1.443485	-3.271079
45	1	2.413424	0.173458	-3.870023
46	64	3.294833	-1.353751	0.281902
47	7	7.732230	-2.009235	-0.714870
48	1	8.252120	-2.794881	-1.087025
49	6	8.470189	-0.812715	-0.305172
50	1	7.857147	-0.294007	0.433234
51	6	8.778078	0.123377	-1.496024
52	1	7.843238	0.343679	-2.019427
53	1	9.441560	-0.390729	-2.198409
54	6	0.128551	0.167492	0.072961

55	1	-0.108391	-0.461279	0.932990
56	1	0.810060	0.946471	0.426317
57	6	-1.162458	0.816796	-0.454629
58	1	-0.950605	1.540342	-1.248816
59	1	-1.846652	0.066532	-0.868437
60	8	-9.483046	-0.248844	-1.911677
61	8	-10.793282	0.955763	2.360428
62	6	-11.109584	-0.633118	-0.229505
63	1	-10.718334	-1.656269	-0.062443
64	1	-10.511112	0.048896	0.377709
65	6	-10.893956	-0.253294	-1.693902
66	1	-11.372607	-0.965294	-2.383496
67	1	-11.319826	0.742623	-1.891906
68	6	-9.139679	-0.064074	-3.278229
69	1	-9.583539	0.863541	-3.671534
70	1	-9.511813	-0.902645	-3.887972
71	6	-12.634824	-0.467398	1.655947
72	1	-12.081773	-1.295820	2.141289
73	1	-13.700306	-0.594319	1.863526
74	6	-12.216995	0.848919	2.299552
75	1	-12.636446	1.690273	1.726821
76	1	-12.628945	0.895660	3.321079
77	6	-10.358779	2.174745	2.947418
78	1	-10.631007	2.209605	4.014474
79	1	-10.829496	3.036450	2.449510
80	8	-8.543520	2.505587	1.435754
81	8	-7.191290	1.241207	-2.833446
82	7	-5.546079	2.799644	-0.693786
83	6	-3.295707	3.498938	0.175199
84	6	-4.730372	3.909023	-0.181697
85	1	-4.672140	4.757153	-0.880461
86	1	-5.177647	4.295562	0.739765
87	6	-6.957969	2.892747	-0.281029
88	1	-7.364291	3.907794	-0.427873
89	1	-7.533357	2.208992	-0.906556
90	6	-7.144218	2.474898	1.176445
91	1	-6.617317	3.151373	1.868408
92	1	-6.740336	1.462245	1.330209
93	6	-8.852853	2.267474	2.804465
94	1	-8.390909	1.329888	3.149429
95	1	-8.471226	3.086807	3.433596
96	6	-5.369083	2.677536	-2.153006
97	1	-5.898392	3.479126	-2.696430
98	1	-4.299494	2.802442	-2.353426
99	6	-5.772016	1.328749	-2.738063
100	1	-5.377645	0.512545	-2.113215
101	1	-5.325916	1.233176	-3.740683
102	6	-7.629880	0.005924	-3.389164
103	1	-7.331239	-0.066069	-4.446562
104	1	-7.181664	-0.841726	-2.849205
105	8	-2.415898	4.348391	0.370033
106	7	-3.121683	2.163982	0.301261
107	1	-3.950573	1.606058	0.106132
108	8	4.486197	-0.401534	2.211395
109	1	5.222876	-0.925790	2.615391
110	1	4.679231	0.566850	2.235723
111	1	9.396095	-1.137429	0.174612
112	6	9.410296	1.411577	-1.020399
113	6	8.603556	2.504703	-0.654293
114	6	10.806434	1.523642	-0.901622
115	6	9.172569	3.684255	-0.180931

116	1	7.522520	2.436568	-0.745813
117	6	11.394139	2.694732	-0.430011
118	1	11.439064	0.688267	-1.186553
119	6	10.562998	3.758975	-0.075625
120	1	8.564799	4.535310	0.100096
121	1	12.468334	2.795033	-0.337591
122	7	11.169442	4.995250	0.418219
123	8	12.402899	5.045617	0.503934
124	8	10.419199	5.929615	0.726926
125	6	-1.868051	1.547403	0.700771
126	1	-1.234217	2.350679	1.086694
127	1	-2.066115	0.846275	1.519994
128	8	4.492363	2.309743	2.157109
129	8	6.022445	-2.346697	3.212842
130	1	4.573634	2.539389	1.187629
131	1	5.045504	2.931777	2.650526
132	1	5.366489	-2.883665	2.702805
133	1	6.878909	-2.537721	2.803761
134	8	1.745972	-1.083140	2.199102
135	1	2.004975	-1.563870	3.031341
136	1	1.795125	-0.113544	2.449401
137	8	2.313693	-2.410068	4.521188
138	8	1.901091	1.543229	2.835242
139	1	2.687975	-3.285877	4.281846
140	1	1.469315	-2.609501	4.952104
141	1	2.793166	1.875709	2.570313
142	1	1.874381	1.653367	3.797191
143	7	-12.511950	-0.504939	0.191084
144	6	-13.327858	-1.605213	-0.347926
145	1	-13.114443	-1.701260	-1.417738
146	1	-13.039188	-2.577319	0.101994
147	6	-14.875892	-1.467523	-0.221456
148	8	-15.354909	-0.458336	0.369104
149	8	-15.508291	-2.426839	-0.748316

E(RTPSSh) = -3642.9420105 Hartree
Zero-point correction = 1.256764
Thermal correction to Energy = 1.337473
Thermal correction to Enthalpy = 1.338417
Thermal correction to Gibbs Free Energy = 1.130445
Sum of electronic and zero-point Energies = -3641.685246
Sum of electronic and thermal Energies = -3641.604538
Sum of electronic and thermal Enthalpies = -3641.603594
Sum of electronic and thermal Free Energies = -3641.811565

Optimized Cartesian coordinates (Å) obtained for the GdL^3(H_2O)·5H_2O system with DFT calculations.

Center Number	Atomic Number	Coordinates (Angstroms) X	Y	Z
1	7	1.884080	-1.074012	-2.815421
2	6	3.120958	-1.775891	-3.249037
3	6	3.117442	-3.261723	-2.911539
4	7	2.927148	-3.552130	-1.466357
5	6	2.413616	-4.939482	-1.313665

6	6	0.911283	-4.996198	-1.561900
7	7	0.145022	-4.189249	-0.580615
8	6	-1.271278	-4.036719	-1.024294
9	6	-1.432277	-3.110684	-2.230299
10	7	-1.011124	-1.709628	-1.949515
11	6	-0.591431	-1.010630	-3.191274
12	6	0.766961	-1.449734	-3.716625
13	6	2.114963	0.397195	-2.852404
14	6	1.711987	1.116664	-1.563094
15	8	0.877244	0.528720	-0.792074
16	8	2.219291	2.245967	-1.345449
17	6	4.193408	-3.395198	-0.727960
18	6	4.467142	-1.930104	-0.434865
19	8	3.514517	-1.133006	-0.265211
20	6	0.181239	-4.860816	0.744766
21	6	1.390901	-4.513698	1.611783
22	8	1.744520	-5.291982	2.502810
23	8	1.939119	-3.343828	1.391425
24	1	3.171035	0.604721	-3.034591
25	1	1.565293	0.857183	-3.684999
26	1	5.041522	-3.866137	-1.245783
27	1	4.074732	-3.874359	0.248608
28	1	0.109625	-5.951578	0.639097
29	1	-0.677628	-4.498473	1.326941
30	1	3.968323	-1.285647	-2.768611
31	1	3.268193	-1.672652	-4.335293
32	1	4.061504	-3.701368	-3.267616
33	1	2.319492	-3.766110	-3.460333
34	1	2.920297	-5.625245	-2.008012
35	1	2.643790	-5.285228	-0.304162
36	1	0.580566	-6.045598	-1.529328
37	1	0.690707	-4.626645	-2.566048
38	1	-1.703986	-5.017317	-1.275625
39	1	-1.821751	-3.634650	-0.170659
40	1	-0.853232	-3.483270	-3.078376
41	1	-2.483291	-3.146277	-2.547474
42	1	-0.557633	0.056121	-2.954538
43	1	-1.331302	-1.154437	-3.992220
44	1	0.782496	-2.534185	-3.847390
45	1	0.917234	-1.008801	-4.714506
46	64	1.109372	-1.750951	-0.185753
47	7	5.744808	-1.548713	-0.309848
48	1	6.472121	-2.226735	-0.502648
49	6	6.137644	-0.191336	0.067658
50	1	5.285151	0.256826	0.579296
51	6	6.534814	0.663423	-1.158375
52	1	5.723714	0.622460	-1.890843
53	1	7.428063	0.234255	-1.623364
54	6	-2.123863	-0.941122	-1.308010
55	1	-2.428258	-1.513249	-0.433277
56	1	-1.681350	-0.013809	-0.930464
57	6	-3.368766	-0.615657	-2.173457
58	1	-3.556741	-1.393002	-2.923911
59	1	-4.237727	-0.623701	-1.506358
60	6	-5.698321	1.723670	-2.902884
61	6	-5.659751	2.032161	-1.402319
62	1	-4.634510	2.104923	-1.028781
63	1	-6.102387	1.183832	-0.868020
64	8	-6.702435	1.980331	-3.585457
65	7	-4.602948	1.144001	-3.475680
66	1	-4.731711	0.912358	-4.454686

67	8	1.752466	-0.351519	1.755151
68	1	2.378852	-0.786705	2.379096
69	1	2.106552	0.549317	1.552096
70	1	6.970938	-0.261028	0.770949
71	6	6.788568	2.099160	-0.759841
72	6	5.745277	3.042401	-0.805547
73	6	8.055467	2.504342	-0.304951
74	6	5.956525	4.360026	-0.405750
75	1	4.762072	2.745789	-1.162949
76	6	8.285995	3.816993	0.098887
77	1	8.870296	1.787246	-0.272738
78	6	7.227483	4.725640	0.042379
79	1	5.164546	5.097873	-0.440032
80	1	9.258070	4.142547	0.447671
81	7	7.459206	6.107745	0.461876
82	8	8.591495	6.415432	0.855041
83	8	6.511336	6.901065	0.403901
84	6	-3.331087	0.763266	-2.861030
85	1	-2.584287	0.791435	-3.657031
86	1	-3.042315	1.527452	-2.132769
87	8	2.540100	2.231609	1.265334
88	8	3.050712	-2.065174	3.391929
89	1	2.342068	2.355559	0.284819
90	1	3.469845	2.474679	1.383649
91	1	2.726089	-2.727989	2.711220
92	1	3.991917	-2.234094	3.537117
93	8	-0.104984	1.255779	4.883369
94	1	0.436226	0.438994	4.902622
95	1	0.346346	1.875670	4.270635
96	8	1.242619	-1.220982	5.298117
97	8	0.903321	3.256889	3.189015
98	1	1.919666	-1.564673	4.670935
99	1	0.531988	-1.877048	5.263354
100	1	1.497493	2.937835	2.467204
101	1	1.446660	3.873109	3.700382
102	6	-5.821582	4.486964	-1.531865
103	1	-6.292838	5.310698	-0.980775
104	1	-6.074333	4.628836	-2.600129
105	6	-4.305377	4.605295	-1.402727
106	6	-2.542767	4.167582	0.139879
107	6	-2.341152	3.643112	1.547200
108	1	-1.271235	3.628576	1.801206
109	1	-2.846507	4.320447	2.254376
110	6	-3.043852	1.909647	2.988612
111	1	-3.545970	2.698421	3.573544
112	1	-2.059869	1.744484	3.450034
113	6	-3.930404	0.675041	3.075002
114	6	-4.443667	-1.622776	2.598246
115	6	-5.330065	-1.789842	1.365369
116	1	-4.693744	-1.969885	0.494405
117	1	-5.972539	-2.673395	1.510171
118	6	-7.327165	-0.512361	1.787813
119	1	-7.139168	0.100417	2.680914
120	1	-7.700633	-1.492493	2.117950
121	6	-8.379391	0.140721	0.909530
122	1	-8.500853	-0.461991	-0.001977
123	1	-9.346025	0.166105	1.436127
124	6	-8.464945	1.911749	-0.673144
125	1	-9.556279	2.049458	-0.624558
126	1	-8.259885	1.166085	-1.455857
127	6	-7.824010	3.240604	-1.043666

128	1	-8.207639	3.501652	-2.044338
129	1	-8.171479	4.017886	-0.348697
130	7	-3.419588	-0.563803	2.461690
131	8	-2.910854	2.338511	1.634000
132	8	-6.136842	-0.664198	1.015066
133	8	-7.951305	1.457801	0.576466
134	1	-1.988958	5.111251	0.013435
135	1	-2.150021	3.444939	-0.592630
136	1	-3.995806	5.611731	-1.725667
137	1	-3.799826	3.887230	-2.068567
138	7	-6.359014	3.240555	-0.987669
139	8	-3.938587	4.360667	-0.053414
140	1	-3.929719	-2.579134	2.738630
141	1	-5.062016	-1.456045	3.497600
142	1	-4.146381	0.519511	4.151445
143	1	-4.880264	0.899334	2.580944
144	6	-2.133954	-0.994126	3.047634
145	1	-1.452112	-0.144025	3.080895
146	1	-2.261041	-1.369597	4.079226
147	6	-1.512170	-2.119936	2.202675
148	8	-0.762545	-1.771394	1.211095
149	8	-1.797883	-3.300554	2.503579

E(RTPSSh) = -3642.9426333 Hartree
Zero-point correction = 1.257939
Thermal correction to Energy = 1.337827
Thermal correction to Enthalpy = 1.338772
Thermal correction to Gibbs Free Energy = 1.138511
Sum of electronic and zero-point Energies = -3641.684694
Sum of electronic and thermal Energies = -3641.604806
Sum of electronic and thermal Enthalpies = -3641.603862
Sum of electronic and thermal Free Energies = -3641.804122

Optimized Cartesian coordinates (Å) obtained for the GdL^5(Gly)(H_2O)·5H_2O system with DFT calculations (simultaneous binding to the metal ion and the crown moiety)

Center Number	Atomic Number	Coordinates (Angstroms) X	Y	Z
1	7	2.749873	-2.949495	-1.218502
2	6	3.713978	-3.705483	-0.372521
3	6	3.052890	-4.367228	0.831980
4	7	2.330810	-3.421285	1.727029
5	6	1.360990	-4.185320	2.560909
6	6	0.100806	-4.531956	1.777833
7	7	-0.634359	-3.332352	1.300764
8	6	-1.628939	-3.723990	0.257929
9	6	-0.990332	-4.064450	-1.088838
10	7	-0.304967	-2.894670	-1.706256
11	6	0.736855	-3.318869	-2.680797
12	6	1.976385	-3.917536	-2.036425
13	6	3.483019	-1.990819	-2.091565
14	6	2.867039	-0.589150	-2.135597
15	8	1.663962	-0.452519	-1.718647
16	8	3.584263	0.342993	-2.576102
17	6	3.278312	-2.706303	2.604963

18	6	3.943415	-1.567227	1.852944
19	8	3.329781	-0.994227	0.922243
20	6	-1.339867	-2.690248	2.441712
21	6	-0.487250	-1.719647	3.262797
22	8	-0.820909	-1.451940	4.420174
23	8	0.528944	-1.178648	2.638239
24	1	4.508587	-1.881255	-1.735508
25	1	3.551661	-2.370718	-3.120294
26	1	4.019534	-3.382395	3.054089
27	1	2.703139	-2.241289	3.411507
28	1	-1.773999	-3.442792	3.113406
29	1	-2.154735	-2.085839	2.024418
30	1	4.491665	-3.016208	-0.044127
31	1	4.215346	-4.489815	-0.959380
32	1	3.823092	-4.912226	1.398175
33	1	2.332723	-5.114187	0.492939
34	1	1.815297	-5.113663	2.936055
35	1	1.109905	-3.577801	3.431949
36	1	-0.554213	-5.145825	2.414307
37	1	0.358417	-5.145868	0.911995
38	1	-2.219063	-4.589095	0.596738
39	1	-2.311457	-2.877288	0.148758
40	1	-0.264425	-4.872627	-0.974532
41	1	-1.775938	-4.453960	-1.751551
42	1	1.013346	-2.430581	-3.255378
43	1	0.336905	-4.060720	-3.385877
44	1	1.695467	-4.756723	-1.396177
45	1	2.614686	-4.332291	-2.831829
46	64	0.961456	-1.461925	0.301705
47	7	5.158554	-1.175250	2.255373
48	1	5.619490	-1.704650	2.985521
49	6	5.862654	-0.032316	1.673547
50	1	5.110115	0.602724	1.204419
51	6	6.920473	-0.467410	0.633557
52	1	6.435243	-1.095852	-0.118987
53	1	7.686484	-1.071768	1.129510
54	6	-1.305063	-2.035690	-2.415583
55	1	-2.149462	-1.908922	-1.736027
56	1	-0.848115	-1.050944	-2.549570
57	6	-1.792789	-2.557916	-3.781569
58	1	-0.994416	-2.449724	-4.522307
59	1	-2.046255	-3.623536	-3.740616
60	6	-5.256195	-2.012748	-3.013585
61	6	-4.884324	-0.933589	-1.989646
62	1	-4.096325	-0.288881	-2.385900
63	1	-4.447054	-1.438569	-1.122991
64	8	-6.356307	-2.584960	-2.994399
65	7	-4.308878	-2.385918	-3.919482
66	1	-4.620508	-3.158654	-4.499576
67	8	1.383299	0.917989	0.779997
68	1	1.656253	1.113438	1.705352
69	1	1.990057	1.408838	0.172048
70	1	6.334770	0.525190	2.486067
71	6	7.551258	0.733022	-0.034219
72	6	6.966775	1.295031	-1.184296
73	6	8.707569	1.325051	0.502487
74	6	7.520496	2.421525	-1.787664
75	1	6.074577	0.846544	-1.614198
76	6	9.276122	2.451565	-0.086656
77	1	9.170519	0.895785	1.386039
78	6	8.669347	2.982839	-1.226193

79	1	7.084692	2.862246	-2.675689
80	1	10.169141	2.914206	0.314638
81	7	9.259152	4.164142	-1.855787
82	8	10.278015	4.648417	-1.347112
83	8	8.709748	4.621700	-2.865764
84	6	-3.026138	-1.789100	-4.302657
85	1	-3.004260	-1.775772	-5.395776
86	1	-2.996743	-0.745190	-3.979973
87	8	2.907169	2.349651	-0.993798
88	8	1.587778	1.074813	3.486134
89	1	3.123757	1.677657	-1.710703
90	1	3.760091	2.671060	-0.666343
91	1	1.140341	0.183751	3.373264
92	1	2.382451	0.926109	4.017617
93	8	-0.758568	3.698238	0.877561
94	1	-0.282772	3.657601	1.737037
95	1	-0.085158	3.922771	0.194442
96	8	0.407340	3.551267	3.423941
97	8	1.054952	4.371600	-1.136895
98	1	0.783656	2.646472	3.531568
99	1	-0.322550	3.589289	4.058093
100	1	1.737267	3.658707	-1.168107
101	1	1.535117	5.143859	-0.804431
102	6	-6.660540	0.630877	-2.612266
103	1	-7.512452	1.164257	-2.179944
104	1	-7.056360	-0.079964	-3.353825
105	6	-5.794130	1.655919	-3.326086
106	6	-4.711974	3.738421	-2.990258
107	6	-4.764239	4.932623	-2.064476
108	1	-4.242949	5.781939	-2.529755
109	1	-5.811891	5.220763	-1.895431
110	6	-4.239561	5.689275	0.102448
111	1	-5.297516	5.910744	0.306258
112	1	-3.777190	6.587356	-0.332274
113	6	-3.520279	5.327241	1.380220
114	1	-2.486446	5.019344	1.170907
115	1	-3.510189	6.209370	2.040385
116	6	-3.611844	3.824534	3.203387
117	1	-3.435512	4.677708	3.877068
118	1	-2.642001	3.353633	2.988823
119	6	-4.540980	2.854655	3.896622
120	1	-4.140594	2.602523	4.889377
121	1	-5.525804	3.324960	4.030210
122	6	-5.674439	0.803621	3.652254
123	1	-6.641338	1.326978	3.680995
124	1	-5.406277	0.518651	4.680022
125	6	-5.780852	-0.439576	2.800614
126	1	-4.800286	-0.922187	2.703496
127	1	-6.473383	-1.141450	3.292236
128	6	-6.462056	-1.219202	0.691616
129	1	-7.216668	-1.882516	1.144303
130	1	-5.523259	-1.783521	0.618791
131	6	-6.977429	-0.780371	-0.667583
132	1	-7.377922	-1.651288	-1.197962
133	1	-7.797731	-0.075968	-0.495892
134	1	-4.356632	1.614133	1.164952
135	1	-4.832587	1.115590	-0.369123
136	1	-4.330696	2.705324	-0.107421
137	7	-4.153667	1.720247	0.155259
138	8	-4.142720	4.600204	-0.822240
139	8	-4.672578	1.668246	3.110438

140	8	-6.274255	-0.071636	1.516623
141	1	-5.136333	4.031990	-3.961777
142	1	-3.673486	3.414788	-3.154128
143	1	-6.365390	2.064236	-4.173724
144	1	-4.875709	1.213709	-3.740231
145	6	-2.726766	1.382517	-0.154551
146	6	-2.360417	-0.039079	0.290463
147	8	-3.251219	-0.753578	0.797496
148	8	-1.144172	-0.377409	0.075426
149	7	-5.974695	-0.071319	-1.505768
150	8	-5.478799	2.692309	-2.402603
151	1	-2.075408	2.123367	0.326504
152	1	-2.590730	1.475942	-1.234721
153	8	-4.238404	4.257807	2.001635

E(RTPSSh) = -3719.8837181 Hartree
Zero-point correction = 1.300331
Thermal correction to Energy = 1.382492
Thermal correction to Enthalpy = 1.383436
Thermal correction to Gibbs Free Energy = 1.178295
Sum of electronic and zero-point Energies = -3718.583387
Sum of electronic and thermal Energies = -3718.501226
Sum of electronic and thermal Enthalpies = -3718.500282
Sum of electronic and thermal Free Energies = -3718.705423

Optimized Cartesian coordinates (Å) obtained for the $GdL^5(Gly)(H_2O)\cdot 5H_2O$ system with DFT calculations (binding to the metal ion)

Center Number	Atomic Number	Coordinates (Angstroms) X	Y	Z
1	7	2.206994	-0.567927	-2.573206
2	6	3.251084	-1.103655	-3.492843
3	6	3.466919	-2.607059	-3.335721
4	7	3.864727	-3.022122	-1.961150
5	6	3.607938	-4.481521	-1.784079
6	6	2.134631	-4.778574	-1.526583
7	7	1.606399	-4.117920	-0.303797
8	6	0.111572	-4.119272	-0.325847
9	6	-0.470095	-3.101889	-1.305404
10	7	-0.136237	-1.699307	-0.931823
11	6	-0.258531	-0.783081	-2.099077
12	6	0.867814	-0.923594	-3.111303
13	6	2.349199	0.908933	-2.437337
14	6	2.183680	1.422576	-1.002630
15	8	1.714649	0.612451	-0.129072
16	8	2.532291	2.605138	-0.764364
17	6	5.295197	-2.743981	-1.718298
18	6	5.511585	-1.290576	-1.335527
19	8	4.591938	-0.645098	-0.776083
20	6	2.084019	-4.827898	0.912034
21	6	3.460332	-4.390292	1.428973
22	8	4.062917	-5.127743	2.212697
23	8	3.882397	-3.212901	1.040942
24	1	3.344197	1.209374	-2.770029
25	1	1.632820	1.435382	-3.082958

26	1	5.924196	-3.033277	-2.571589
27	1	5.607836	-3.328761	-0.847772
28	1	2.091166	-5.915148	0.759384
29	1	1.380399	-4.606788	1.721014
30	1	4.180673	-0.568023	-3.298232
31	1	2.985748	-0.904688	-4.541621
32	1	4.225666	-2.929544	-4.063995
33	1	2.548319	-3.141171	-3.587068
34	1	3.934303	-5.043747	-2.670425
35	1	4.211780	-4.827039	-0.943530
36	1	2.004170	-5.868512	-1.453236
37	1	1.534575	-4.449996	-2.377712
38	1	-0.265891	-5.119432	-0.586253
39	1	-0.218395	-3.887482	0.690839
40	1	-0.096490	-3.285205	-2.315782
41	1	-1.557249	-3.250074	-1.352975
42	1	-0.275053	0.235318	-1.702473
43	1	-1.206192	-0.947944	-2.630264
44	1	0.911896	-1.951660	-3.478143
45	1	0.634032	-0.292149	-3.981903
46	64	2.447671	-1.634675	-0.066450
47	7	6.719510	-0.760634	-1.554469
48	1	7.409914	-1.316172	-2.045192
49	6	7.088392	0.587490	-1.120037
50	1	6.416499	0.856220	-0.303680
51	6	6.980168	1.621523	-2.263971
52	1	5.980473	1.555845	-2.703087
53	1	7.705110	1.373440	-3.045414
54	6	-1.041013	-1.209780	0.158257
55	1	-1.013342	-1.949374	0.960738
56	1	-0.590029	-0.289051	0.541975
57	6	-2.501346	-0.938448	-0.241164
58	1	-2.562601	-0.150993	-0.998247
59	1	-2.973824	-1.835973	-0.657075
60	6	-5.048050	1.053002	0.100430
61	6	-6.532957	1.223244	-0.252317
62	1	-7.010898	1.798753	0.547217
63	1	-7.040725	0.252712	-0.275378
64	8	-4.223688	1.964475	-0.068554
65	7	-4.696529	-0.152483	0.622653
66	1	-5.391604	-0.884389	0.684997
67	8	3.545152	-0.318117	1.772736
68	1	4.409815	-0.676146	2.088937
69	1	3.615454	0.668293	1.674206
70	1	8.109994	0.550231	-0.734598
71	6	7.220069	3.022339	-1.748900
72	6	6.150248	3.786528	-1.247199
73	6	8.515838	3.566377	-1.728142
74	6	6.364123	5.064164	-0.736054
75	1	5.141071	3.382959	-1.262544
76	6	8.749908	4.842148	-1.221131
77	1	9.348045	2.987955	-2.118012
78	6	7.665206	5.571489	-0.730940
79	1	5.549957	5.665874	-0.351590
80	1	9.742714	5.274046	-1.203525
81	7	7.898789	6.913990	-0.198845
82	8	9.057154	7.349175	-0.203052
83	8	6.926655	7.548146	0.230058
84	6	-3.316079	-0.470391	0.976335
85	1	-2.870119	0.436513	1.395269
86	1	-3.333078	-1.237613	1.755525

87	8	3.443288	2.388262	1.709054
88	8	5.453844	-1.940100	2.723627
89	1	2.992711	2.576636	0.826828
90	1	4.279781	2.876760	1.692150
91	1	4.987673	-2.595419	2.120547
92	1	6.403170	-2.007118	2.548528
93	8	4.917643	1.363207	5.520286
94	1	4.802040	0.394355	5.483768
95	1	4.084373	1.706846	5.143367
96	8	4.369554	-1.459870	5.152525
97	8	2.440454	2.125572	4.227213
98	1	4.499364	-2.154072	5.814074
99	1	4.857143	-1.748663	4.338450
100	1	2.712983	2.346922	3.300591
101	1	1.895999	2.863160	4.536500
102	6	-6.489013	3.331081	-1.586267
103	1	-7.030125	3.732096	-2.453745
104	1	-5.414634	3.512925	-1.753959
105	6	-6.880437	4.124150	-0.349445
106	6	-8.668215	4.560504	1.133438
107	6	-10.120044	4.218095	1.398557
108	1	-10.480293	4.815788	2.251030
109	1	-10.732116	4.477518	0.520814
110	6	-11.558590	2.438902	1.968635
111	1	-12.232619	2.735077	1.150085
112	1	-11.912103	2.926118	2.891396
113	6	-11.611717	0.935112	2.146993
114	1	-10.841042	0.614800	2.865128
115	1	-12.594923	0.659017	2.560326
116	6	-11.521715	-1.102491	0.958726
117	1	-12.506222	-1.390731	1.360466
118	1	-10.749928	-1.518626	1.624670
119	6	-11.369532	-1.682919	-0.432801
120	1	-11.662368	-2.744912	-0.416646
121	1	-12.043900	-1.156393	-1.125538
122	6	-9.826517	-1.979816	-2.191789
123	1	-10.433290	-1.373760	-2.882271
124	1	-10.131628	-3.032037	-2.307469
125	6	-8.358671	-1.851135	-2.543560
126	1	-7.751143	-2.385160	-1.796473
127	1	-8.177667	-2.318009	-3.524638
128	6	-6.617297	-0.273505	-2.757735
129	1	-6.293819	-0.666438	-3.734613
130	1	-6.051742	-0.823015	-1.988275
131	6	-6.300288	1.216303	-2.706651
132	1	-5.203318	1.317646	-2.817820
133	1	-6.753806	1.708253	-3.576038
134	1	2.296854	-0.639865	3.091160
135	1	2.717206	-1.030106	4.641662
136	1	1.870399	0.393881	4.317966
137	7	1.944095	-0.615033	4.075543
138	8	-10.221086	2.827336	1.679967
139	8	-10.016518	-1.545696	-0.850544
140	8	-8.012225	-0.471681	-2.569205
141	1	-8.566643	5.653319	1.038477
142	1	-8.046608	4.234903	1.981953
143	1	-6.686162	5.191354	-0.541278
144	1	-6.258656	3.828148	0.509227
145	6	0.673294	-1.387296	4.155548
146	6	0.525369	-2.313863	2.932539
147	8	-0.309091	-3.230056	2.991762

148	8	1.269221	-2.010652	1.929892
149	7	-6.806764	1.898910	-1.514556
150	8	-8.257840	3.908980	-0.061782
151	1	-0.165392	-0.687586	4.159664
152	1	0.646413	-1.963999	5.079348
153	8	-11.411455	0.313823	0.883003

E(RTPSSh) = -3719.8773801 Hartree
Zero-point correction = 1.297693
Thermal correction to Energy = 1.380661
Thermal correction to Enthalpy = 1.381605
Thermal correction to Gibbs Free Energy = 1.167646
Sum of electronic and zero-point Energies = -3718.579687
Sum of electronic and thermal Energies = -3718.496720
Sum of electronic and thermal Enthalpies = -3718.495775
Sum of electronic and thermal Free Energies = -3718.709734

Optimized Cartesian coordinates (Å) obtained for the GdL^5(Gly)$(H_2O)_2\cdot 4H_2O$ system with DFT calculations

Center Number	Atomic Number	Coordinates (Angstroms) X	Y	Z
1	7	-3.144315	-0.376821	2.138700
2	6	-4.130788	-0.835648	3.156633
3	6	-4.377424	-2.341302	3.095997
4	7	-4.873764	-2.819527	1.774435
5	6	-4.664340	-4.292693	1.659434
6	6	-3.215079	-4.644548	1.343711
7	7	-2.734649	-4.084034	0.050356
8	6	-1.241035	-4.114243	0.010420
9	6	-0.597139	-3.048877	0.895669
10	7	-0.918603	-1.668959	0.443732
11	6	-0.709422	-0.676292	1.532534
12	6	-1.777122	-0.718251	2.615552
13	6	-3.278517	1.088162	1.902221
14	6	-3.130756	1.486672	0.428389
15	8	-2.707847	0.586093	-0.381994
16	8	-3.439797	2.651567	0.088538
17	6	-6.311883	-2.515782	1.615888
18	6	-6.516272	-1.077744	1.171544
19	8	-5.625492	-0.498070	0.502802
20	6	-3.286531	-4.865178	-1.085918
21	6	-4.619729	-4.352024	-1.644592
22	8	-5.150906	-5.006126	-2.560835
23	8	-5.066154	-3.230061	-1.179963
24	1	-4.268108	1.415525	2.225155
25	1	-2.548952	1.654813	2.497174
26	1	-6.885004	-2.742581	2.525409
27	1	-6.700477	-3.138027	0.803887
28	1	-3.389548	-5.928717	-0.833292
29	1	-2.582703	-4.805357	-1.921066
30	1	-5.062877	-0.292783	2.997046
31	1	-3.791101	-0.585467	4.172350
32	1	-5.088854	-2.613303	3.889338
33	1	-3.450533	-2.876233	3.312313

34	1	-4.956352	-4.800520	2.589360
35	1	-5.319991	-4.659186	0.868544
36	1	-3.113397	-5.739631	1.338619
37	1	-2.562582	-4.274878	2.137182
38	1	-0.866397	-5.103782	0.309160
39	1	-0.948138	-3.947979	-1.028495
40	1	-0.931582	-3.158130	1.929750
41	1	0.486976	-3.222764	0.908691
42	1	-0.695177	0.309618	1.062172
43	1	0.264381	-0.827743	2.018886
44	1	-1.814253	-1.714447	3.062435
45	1	-1.480533	-0.028339	3.420106
46	64	-3.564576	-1.582319	-0.271745
47	7	-7.683865	-0.493168	1.457450
48	1	-8.350396	-0.998669	2.028912
49	6	-8.047371	0.841381	0.977327
50	1	-7.414084	1.053796	0.115031
51	6	-7.859557	1.928046	2.060365
52	1	-6.837240	1.870750	2.445569
53	1	-8.541247	1.731419	2.893730
54	6	-0.071300	-1.276823	-0.727299
55	1	-0.200917	-2.044942	-1.491992
56	1	-0.503742	-0.354303	-1.124680
57	6	1.428197	-1.067151	-0.454270
58	1	1.594016	-0.210260	0.206809
59	1	1.870559	-1.945012	0.031375
60	6	4.188451	0.617720	-1.601489
61	6	5.709691	0.581746	-1.400952
62	1	6.166295	0.612914	-2.396292
63	1	6.022759	-0.363881	-0.943475
64	8	3.551403	1.664443	-1.792475
65	7	3.600537	-0.607693	-1.579207
66	1	4.172256	-1.413854	-1.361042
67	8	-4.698334	-0.479674	-2.152236
68	1	-5.627726	-0.748691	-2.364036
69	1	-4.558903	0.485814	-2.307617
70	1	-9.088549	0.809775	0.647733
71	6	-8.112657	3.304825	1.489170
72	6	-7.073429	4.014175	0.859306
73	6	-9.394125	3.879224	1.545840
74	6	-7.302688	5.268601	0.300051
75	1	-6.075745	3.585828	0.809559
76	6	-9.643444	5.132485	0.992062
77	1	-10.202424	3.342259	2.033181
78	6	-8.588572	5.807958	0.375881
79	1	-6.511689	5.828230	-0.183456
80	1	-10.625126	5.587596	1.032965
81	7	-8.837841	7.126569	-0.206567
82	8	-9.982811	7.589840	-0.131420
83	8	-7.891753	7.713417	-0.746632
84	6	2.170278	-0.809101	-1.776350
85	1	1.791379	0.096314	-2.256743
86	1	2.027532	-1.650146	-2.463416
87	8	-3.789716	2.046217	-2.535661
88	8	-6.948303	-1.847461	-2.609862
89	1	-3.615154	2.391741	-1.614019
90	1	-4.181448	2.768192	-3.047229
91	1	-6.429002	-2.525468	-2.110593
92	1	-7.728975	-1.674297	-2.064035
93	8	-2.378981	-2.082562	-2.389183
94	1	-2.926612	-2.525954	-3.092498

95	1	-2.145115	-1.191021	-2.783671
96	8	-3.763295	-3.361437	-4.369423
97	8	-1.761535	0.347376	-3.410874
98	1	-4.362157	-4.012868	-3.943526
99	1	-3.126526	-3.897014	-4.865519
100	1	-2.430330	0.998671	-3.086707
101	1	-1.872160	0.341140	-4.373132
102	6	6.359961	2.957265	-1.362585
103	1	6.573879	3.754631	-0.643909
104	1	5.401577	3.185179	-1.845779
105	6	7.465889	2.975290	-2.405443
106	6	9.814237	3.026551	-2.658390
107	6	11.098139	3.161565	-1.870648
108	1	11.936544	3.311425	-2.566027
109	1	11.040844	4.033977	-1.204045
110	6	12.534903	2.022564	-0.381037
111	1	12.534616	2.880775	0.306062
112	1	13.376716	2.137565	-1.078852
113	6	12.702874	0.738444	0.400179
114	1	12.637151	-0.129085	-0.273182
115	1	13.698900	0.732956	0.868137
116	6	11.811320	-0.439345	2.253928
117	1	12.813882	-0.466960	2.706519
118	1	11.661798	-1.375844	1.696293
119	6	10.781575	-0.311047	3.354594
120	1	10.942799	-1.103477	4.099739
121	1	10.891767	0.661887	3.854887
122	6	8.454864	-0.275395	3.782231
123	1	8.558474	0.700007	4.279583
124	1	8.550980	-1.063338	4.543322
125	6	7.103147	-0.380331	3.113243
126	1	7.040204	-1.301296	2.519668
127	1	6.324842	-0.398009	3.892803
128	6	5.643661	0.739096	1.647923
129	1	4.865781	0.868546	2.417468
130	1	5.476568	-0.226795	1.155067
131	6	5.548698	1.899880	0.668506
132	1	4.487328	2.118018	0.477442
133	1	5.983079	2.781004	1.151591
134	1	8.997927	0.240729	1.014278
135	1	7.995907	0.886042	-0.178269
136	1	9.651812	1.213545	-0.188235
137	7	8.948219	0.485880	0.011874
138	8	11.304396	1.972019	-1.106103
139	8	9.474807	-0.429508	2.792116
140	8	6.930619	0.751250	2.263396
141	1	9.709536	3.903420	-3.315454
142	1	9.847661	2.127727	-3.292387
143	1	7.369404	3.896601	-3.000538
144	1	7.391078	2.128665	-3.105045
145	6	9.247104	-0.712796	-0.830670
146	6	8.306386	-1.917577	-0.565607
147	8	7.277528	-1.699113	0.138922
148	8	8.675992	-2.983930	-1.114293
149	7	6.279008	1.685693	-0.602551
150	8	8.727882	2.954831	-1.741941
151	1	10.282282	-0.998874	-0.639609
152	1	9.171072	-0.406499	-1.876943
153	8	11.683535	0.687782	1.392565

E(RTPSSh) = -3719.8762646 Hartree

```
Zero-point correction = 1.299463
Thermal correction to Energy = 1.382211
Thermal correction to Enthalpy = 1.383155
Thermal correction to Gibbs Free Energy = 1.171587
Sum of electronic and zero-point Energies = -3718.576801
Sum of electronic and thermal Energies = -3718.494053
Sum of electronic and thermal Enthalpies = -3718.493109
Sum of electronic and thermal Free Energies = -3718.704677
```

Optimized Cartesian coordinates (Å) obtained for the GdL^{4}(H$_2$O)$_2$·4H$_2$O system with DFT calculations

Center Number	Atomic Number	Coordinates (Angstroms) X	Y	Z
1	7	1.938905	-0.686403	-2.880625
2	6	3.033560	-1.531360	-3.426113
3	6	2.809549	-3.021723	-3.192287
4	7	2.647081	-3.394602	-1.762367
5	6	1.965413	-4.714470	-1.677801
6	6	0.458324	-4.574593	-1.859515
7	7	-0.171860	-3.721963	-0.819963
8	6	-1.553287	-3.335479	-1.231823
9	6	-1.581864	-2.308337	-2.364756
10	7	-0.989466	-1.001369	-1.970331
11	6	-0.512106	-0.237714	-3.151882
12	6	0.759162	-0.792959	-3.773099
13	6	2.394859	0.726483	-2.774230
14	6	2.078288	1.385722	-1.427869
15	8	1.178354	0.846149	-0.695971
16	8	2.721541	2.423028	-1.126846
17	6	3.956153	-3.462601	-1.087703
18	6	4.437673	-2.072718	-0.709867
19	8	3.610272	-1.171899	-0.437233
20	6	-0.215005	-4.458156	0.469860
21	6	1.071255	-4.372977	1.305893
22	8	1.298225	-5.251236	2.142883
23	8	1.824673	-3.327041	1.094224
24	1	3.475689	0.777861	-2.917789
25	1	1.950270	1.342444	-3.568470
26	1	4.707140	-3.998358	-1.686544
27	1	3.819322	-3.997787	-0.143270
28	1	-0.476456	-5.513571	0.317607
29	1	-0.990355	-3.997655	1.085736
30	1	3.967821	-1.214717	-2.961608
31	1	3.149602	-1.370671	-4.509188
32	1	3.651443	-3.574226	-3.636815
33	1	1.913285	-3.348732	-3.723536
34	1	2.352881	-5.406037	-2.440006
35	1	2.190431	-5.155823	-0.705673
36	1	0.003745	-5.576940	-1.854398
37	1	0.245070	-4.138566	-2.837922
38	1	-2.128611	-4.221840	-1.538672
39	1	-2.025032	-2.904084	-0.346441
40	1	-1.043363	-2.684277	-3.237699

41	1	-2.625131	-2.185524	-2.688298
42	1	-0.327226	0.783952	-2.809287
43	1	-1.283276	-0.194042	-3.934865
44	1	0.620337	-1.844429	-4.034222
45	1	0.948445	-0.258694	-4.717566
46	64	1.136855	-1.493126	-0.289822
47	7	5.760323	-1.875579	-0.629135
48	1	6.377298	-2.629504	-0.905487
49	6	6.355322	-0.607965	-0.207879
50	1	5.603543	-0.080336	0.380315
51	6	6.793348	0.254626	-1.414067
52	1	5.936813	0.379974	-2.082733
53	1	7.575852	-0.273038	-1.968790
54	6	-1.987572	-0.164445	-1.230622
55	1	-2.331361	-0.749051	-0.376196
56	1	-1.424015	0.678906	-0.816374
57	6	-3.196168	0.357043	-2.056276
58	1	-3.323024	-0.207111	-2.988114
59	1	-4.111464	0.196222	-1.478530
60	6	-5.488906	2.701733	-2.682097
61	6	-5.795120	2.447320	-1.203572
62	1	-4.885662	2.464180	-0.595788
63	1	-6.186192	1.427734	-1.111444
64	8	-6.346452	3.163529	-3.450956
65	7	-4.244349	2.377505	-3.139807
66	1	-4.129743	2.503195	-4.139364
67	8	2.035591	-0.388310	1.708647
68	1	2.661102	-0.948377	2.228196
69	1	2.455339	0.499272	1.587096
70	1	7.211097	-0.829481	0.434252
71	6	7.291479	1.608867	-0.964660
72	6	6.401487	2.693555	-0.858224
73	6	8.640163	1.795209	-0.614555
74	6	6.843406	3.937362	-0.413373
75	1	5.356330	2.566511	-1.129966
76	6	9.100648	3.031088	-0.167932
77	1	9.337156	0.966712	-0.699020
78	6	8.189897	4.085167	-0.074152
79	1	6.171472	4.782631	-0.331126
80	1	10.137770	3.188840	0.100423
81	7	8.663655	5.388915	0.389995
82	8	9.860525	5.503646	0.682875
83	8	7.844307	6.312972	0.467899
84	6	-3.098605	1.856116	-2.391619
85	1	-2.215247	2.059351	-3.001464
86	1	-2.977555	2.429909	-1.466319
87	8	3.065236	2.142542	1.471678
88	8	3.354881	-2.346386	3.025540
89	1	2.859721	2.383332	0.514923
90	1	4.027962	2.207891	1.556295
91	1	2.860609	-2.913118	2.365458
92	1	4.294157	-2.557724	2.930996
93	8	0.508865	0.895127	4.823790
94	1	1.091021	0.097446	4.955177
95	1	1.071362	1.620497	4.448966
96	8	1.640781	-1.533868	5.134498
97	8	1.896714	3.012309	3.791496
98	1	2.379551	-1.778007	4.541369
99	1	0.845690	-1.853516	4.623502
100	1	2.284960	2.770219	2.915633
101	1	2.666178	3.174905	4.356373

102	6	-6.348596	4.755066	-0.534326
103	1	-7.025991	5.265711	0.162269
104	1	-6.455301	5.258153	-1.514107
105	6	-4.908531	4.962172	-0.074077
106	6	-3.352015	4.047012	1.489451
107	6	-3.321577	2.985335	2.571235
108	1	-2.296088	2.856067	2.946800
109	1	-3.954053	3.313701	3.411162
110	6	-4.190555	0.854907	3.063348
111	1	-4.934936	1.335841	3.719457
112	1	-3.314981	0.599358	3.672489
113	6	-4.844586	-0.383376	2.471928
114	6	-4.793995	-2.449436	1.265035
115	6	-5.365227	-2.175878	-0.128028
116	1	-4.541484	-1.941882	-0.806845
117	1	-5.852469	-3.098406	-0.481737
118	6	-7.593155	-1.329060	0.228489
119	1	-7.661645	-1.126777	1.306571
120	1	-7.872271	-2.378646	0.057214
121	6	-8.548565	-0.435860	-0.541814
122	1	-8.406995	-0.614393	-1.617281
123	1	-9.588003	-0.691657	-0.285180
124	6	-8.615013	1.814503	-1.292150
125	1	-9.703422	1.827119	-1.456935
126	1	-8.142571	1.475370	-2.226007
127	6	-8.160800	3.230256	-0.969462
128	1	-8.400433	3.835148	-1.860507
129	1	-8.761274	3.625704	-0.138031
130	7	-3.954330	-1.364857	1.816588
131	8	-3.819716	1.766653	2.031195
132	8	-6.271220	-1.080609	-0.252692
133	8	-8.273518	0.927092	-0.228091
134	1	-2.862344	4.963601	1.853858
135	1	-2.797128	3.698406	0.604522
136	1	-4.715990	6.041457	0.028179
137	1	-4.202413	4.581792	-0.829384
138	7	-6.751923	3.349655	-0.581368
139	8	-4.714567	4.282902	1.157945
140	1	-4.169985	-3.341388	1.145658
141	1	-5.605541	-2.724399	1.964787
142	1	-5.393743	-0.875448	3.299646
143	1	-5.580772	-0.063774	1.730799
144	6	-2.943920	-1.898020	2.754023
145	1	-3.233052	-1.731666	3.804230
146	1	-2.882881	-2.986186	2.638507
147	15	-1.176596	-1.368050	2.610515
148	8	-1.135718	0.195110	2.992097
149	8	-0.733040	-1.525652	1.130642
150	8	-0.456469	-2.230651	3.644712
151	1	-0.460060	0.444382	3.733249

E(RTPSSh) = -4022.1079927 Hartree
Zero-point correction = 1.270382
Thermal correction to Energy = 1.350220
Thermal correction to Enthalpy = 1.351164
Thermal correction to Gibbs Free Energy = 1.151816
Sum of electronic and zero-point Energies = -4020.837611
Sum of electronic and thermal Energies = -4020.757773
Sum of electronic and thermal Enthalpies = -4020.756828
Sum of electronic and thermal Free Energies = -4020.956176

6. References

Angelovski, G., Fouskova, P., Mamedov, I., Canals, S., Toth, E., and Logothetis, N.K. (2008). Smart magnetic resonance imaging agents that sense extracellular calcium fluctuations. *ChemBioChem* 9(11)**,** 1729-1734. doi: 10.1002/cbic.200800165.

Gündüz, S., Nitta, N., Vibhute, S., Shibata, S., Maier, M.E., Logothetis, N.K., et al. (2015). Dendrimeric calcium-responsive MRI contrast agents with slow *in vivo* diffusion. *Chem. Commun.* 51(14)**,** 2782-2785. doi: 10.1039/c4cc07540d.
